# Recent Developments in Cellular Immunotherapy for HSCT-Associated Complications

**DOI:** 10.3389/fimmu.2016.00500

**Published:** 2016-11-14

**Authors:** Monica Reis, Justyna Ogonek, Marsela Qesari, Nuno M. Borges, Lindsay Nicholson, Liane Preußner, Anne Mary Dickinson, Xiao-nong Wang, Eva M. Weissinger, Anne Richter

**Affiliations:** ^1^Haematological Sciences, Institute of Cellular Medicine, Newcastle University, Newcastle upon Tyne, UK; ^2^Transplantation Biology, Department of Hematology, Hemostasis, Oncology and Stem Cell Transplantation, Hannover Medical School, Hannover, Germany; ^3^Alcyomics Ltd., Newcastle upon Tyne, UK; ^4^Miltenyi Biotec GmbH, Bergisch Gladbach, Germany

**Keywords:** mesenchymal stromal cells, immunomodulation, extracellular vesicles, infection, adoptive transfer, chimeric antigen receptor, T cells, cell manufacture

## Abstract

Allogeneic hematopoietic stem cell transplantation is associated with serious complications, and improvement of the overall clinical outcome of patients with hematological malignancies is necessary. During the last decades, posttransplant donor-derived adoptive cellular immunotherapeutic strategies have been progressively developed for the treatment of graft-versus-host disease (GvHD), infectious complications, and tumor relapses. To date, the common challenge of all these cell-based approaches is their implementation for clinical application. Establishing an appropriate manufacturing process, to guarantee safe and effective therapeutics with simultaneous consideration of economic requirements is one of the most critical hurdles. In this review, we will discuss the recent scientific findings, clinical experiences, and technological advances for cell processing toward the application of mesenchymal stromal cells as a therapy for treatment of severe GvHD, virus-specific T cells for targeting life-threating infections, and of chimeric antigen receptors-engineered T cells to treat relapsed leukemia.

## Introduction

The medical need for improved therapeutic options to successfully treat patients with hematologic malignancies is high. Allogeneic hematopoietic stem cell transplantation (HSCT) is the only curative treatment for patients with hematologic malignancies, but the success of the therapy is limited by several severe side effects. One major obstacle with the highest transplant-related mortality rate is the recurrence of the underlying disease, due to failure in effective eradication of malignant cells by the reconstituted allogeneic immune system, mediated largely by T cells. The leading cause of non-relapse mortality is graft-versus-host disease (GvHD), an inflammatory immune reaction against healthy tissue of the patient, induced by donor-derived T cells and triggered by major and minor histocompatibility antigen differences between HSCT recipient and donor. Due to immunosuppressive treatment of the patient for prophylaxis and posttransplant therapy of GvHD, the appearance of life-threatening opportunistic infections is responsible for a substantial rate of non-relapse mortality. Thus, one of the biggest challenges for an effective treatment with allogeneic HSCT is maintaining the balance between tolerance of the host, elimination of the malignancy, and protection against infections.

Engineering of the allograft itself is one possible strategy to reduce the risk for development of GvHD and concomitantly remain the favorable immune reaction toward the tumor and infectious pathogens. The incidence and severity of GvHD can be reduced by *ex vivo* T cell removal either achieved *via* CD34^+^ hematopoietic stem cell enrichment or active depletion of T cells, but these approaches have been associated with the risk for occurrence of graft rejection, relapse, and infections due to the missing T cells. However, for matched sibling donor transplantation in acute myeloid leukemia, it has been shown recently that *ex vivo* T cell depletion can reduce the incidence of chronic GvHD significantly without affecting the relapse rate (1, 2). The most novel procedures in graft manipulation aim for the elimination of potential alloreactive T cells only, allowing antiviral and antitumor T cells to remain in the transplant supporting tumor elimination and providing protection against infections (3–8).

Another strategy to control allogeneic HSCT-related complications is the adaptive transfer of *ex vivo* selected donor-derived immune cell populations after transplantation. At first, donor lymphocyte infusions (DLI) were established to prevent and treat relapses, but, subsequently, controlling infections became an important matter for concern (9, 10). DLI contain allogeneic T cells and are therefore associated with an increased risk for the onset of GvHD. These observations initiated the development of several adoptive therapies with selected immune cell populations depleted of alloreactive cells. Strategies that are followed include the adoptive therapy of regulatory T cells (Tregs) and mesenchymal stromal cells (MSCs) for treatment of GvHD, dendritic cell (DC) vaccination and natural killer (NK) cell transfer to support antitumor responses, as well as application of T cells to control infections or to induce antitumor responses (11–13).

Despite the differences in cell type and the underlying medical problem, which require specific considerations during the translational phase, various hurdles are common for all cellular immunotherapies. At present, a variety of clinical protocols, including cell manufacturing processes, have been generated for each of the three therapeutic approaches and reached a stage of evaluation within clinical trials. However, the obstacles, prior to clinical application which remain, include the establishment of standardized clinical protocols and understanding the therapeutic mechanisms. Nevertheless, the promising and beneficial clinical outcomes of early-phase clinical studies, the enormous achievements in scientific understanding of immune interventions, and the innovative technical advances in cell manipulation and processing has led to a huge growth in interest in cellular immunotherapy, especially in the area of hematological diseases. To offer these new therapeutic options as standard-of-care treatments for all patients, various aspects have to be considered for the implementation into clinical practice, in particular with regard to the cell manufacturing. Cell-processing protocols, often developed in research laboratories using tools and technologies available or suitable for research application only, need to be process engineered to good manufacturing practice (GMP) prior to clinical application.

This review will discuss the challenges and recent progresses made toward clinical application of MSCs for the management of GvHD, antiviral T cells for the treatment of opportunistic viral infections, and chimeric antigen receptors (CAR)-engineered T cells as an adoptive therapy for leukemia relapses. These three examples allow us to not only to highlight technological and clinical advances of the individual therapy but also discuss general aspects of translation, especially with regard to cell processing.

## Clinical Application with Mesenchymal Stromal Cells for the Management of GvHD

Mesenchymal stromal cells are multipotent progenitor cells, which can be acquired from various adult tissues, primarily bone marrow (BM) (14). Their immunomodulatory property has empowered them to play an important role as a cellular therapy for GvHD (15). GvHD is a frequent and potentially life-threatening complication after allogeneic HSCT, affecting 40–60% of patients, and a leading cause of non-relapse mortality (16, 17). Despite significant advances in the understanding of GvHD pathogenesis and the development of transplantation medicine, corticosteroids remain the first-line treatment of GvHD, but with only an approximately 50% response rate. Patients who fail the standard steroid treatment have an overall survival rate of only 5–30% (18–20). Apart from the low response rate, steroid treatment also bears the risk of increased leukemia relapse and opportunistic infections. To improve the efficacy of GvHD management, several cellular immunotherapies have been developed using MSCs as well as DCs and Tregs (17, 21, 22).

### Lessons Learned from Recent Clinical Trials

Since the first case report in which infusion of haploidentical MSCs showed a beneficial outcome in the treatment of severe treatment-refractory acute GvHD (aGvHD) (23), an increasing number of clinical trials have been conducted to evaluate the effect of MSC infusion on GvHD for over a decade (17, 24, 25). The outcome of early clinical trials has been well reviewed. This article mainly collates recent clinical studies, reported between 2010 and 2015, on the prophylactic and therapeutic use of MSCs for aGvHD. The relevant information is summarized in Table 1 (26–29) and Table 2 (15, 30–38), respectively.

**Table 1:** **Prophylactic use of MSCs to prevent GvHD**.

MSCs	HSCs	MSC group	Ctrl group	Observation on GvHD incidence/severity	Reference
UCB	BM, PBSC	21	None	9 of 21 patients developed aGvHD (II–IV)	(27)
0.5 × 10^6^/kg	Haploidentical				
Single dose	Without TCD				
UCB	BM, PBSC	50	None	12 of 50 patients developed aGvHD (II–IV)	(26)
0.5 × 10^6^/kg	Haploidentical				
Single dose					
BM-PL of HSC donor	BM	19	18	1 of 19 patients had aGvHD in MSC group	(28)
0.9–1.3 × 10^6^/kg	Donor type NR		Randomized	6 of 18 patients had aGvHD (II–IV) in Ctrl group	
Single dose					
BM, third party	PBSC	20	16	9 of 20 patients had aGvHD (II–IV) in MSC group	(29)
0.9–1.3 × 10^6^/kg	MMR or MMU		Historic	9 of 16 patients had aGvHD (II–IV) in Ctrl group	
Single dose					

*BM, bone marrow; PBSC, peripheral blood stem cells; UCB, umbilical cord blood; HSCs, hematopoietic stem cells; NR, not reported; BM-PL, platelet lysate expanded MSC; MMR, HLA-mismatch related donors; MMU, HLA-mismatched unrelated donors; TCD, T cell depletion*.

**Table 2:** **Therapeutic use of MSC infusion for steroid-resistant/refractory aGvHD**.

MSCs	HSCs	No. Pts	Clinical outcome	Reference
BM, third party	BM, PBSC, CUB	28	CR: 61%	(15)
1 × 10^6^/kg	HLA identical		OR: 75%	
2–8 infusions	Haploidentical			
	HLA-mismatched			
BM-PL, third party	BM, PBSC, UCB	40	CR: 27.5%	(30)
1.5 × 10^6^/kg	HLA identical		OR: 67.5%	
1–5 infusions	Haploidentical			
	HLA-mismatched			
BM-PL, third party	NR	25	CR: 46%	(31)
1.1 × 10^6^/kg			OR: 71%	
2–4 infusions				
BM, third party	BM, PBSC, UCB, DLI	75	CR: NR	(35)
2 × 10^6^/kg	HLA-matched		OR: 61.3%	
8–12 infusions	HLA-mismatched			
BM, third party *n* = 34	BM, PBSC, UCB, DLI	37	CR: 65%	(36)
1–2 × 10^6^/kg	HLA identical, MUD		OR: 86%	
1–13 infusions	Haploidentical			
BM, third party	BM, PBSC, UCB	50	CR: 34%	(37)
1.1 × 10^6^/kg	HLA identical, MUD		IR: 66%	
1–4 infusions	Haploidentical, UCB			
BM, third party	PBSC	12	CR: 58.3%	(38)
1.7–2.3 × 10^6^/kg	MUD		OR: 91.7%	
2–8 infusion				
BM-AS/AS + PL	BM, PBSC	10	CR: 10%	(32)
haplo- & RD	HLA-matched		OR: 70%	
1–2 × 10^6^/kg	HLA-mismatched			
1–4 infusions				
BM, third party	BM, PBSC, UCB	12	CR: 58%	(34)
8 × 10^6^/kg *n* = 2	HLA-matched		OR: 75%	
2 × 10^6^/kg *n* = 10	HLA-mismatched			
8–12 infusions				
BM-PL, third party	BM, PBSC, UCB	11	CR: 23.8%	(33)
1.2 × 10^6^/kg	HLA-matched		OR: 71.4%	
1–5 infusions	HLA-mismatched			

*No. Pts, number of patients; BM, bone marrow; PBSC, peripheral blood stem cells; UCB, umbilical cord blood; DLI, donor lymphocyte infusions; MUD, HLA-matched unrelated donor; RD, related donors; HSC, hematopoietic stem cells; NR, not reported; BM-PL, platelet lysate expanded MSC; BM-AS, human serum expanded MSC; CR, complete response; OR, overall response; IR, initial response*.

These reports have shown encouraging results indicative of positive steps taken toward the development of a more refined MSC therapy, although significant improvements are still needed. First, recent clinical studies have shown a clear trend toward replacing fetal calf serum (FCS) with human platelet lysate (hPL) to generate MSCs. Until the first clinical trial utilizing hPL-expanded MSCs to treat aGvHD being reported in 2009 (25), all clinical trials in the HSCT setting were performed using MSCs expanded in FCS-containing medium, a condition no longer accepted under current regulatory GMP requirements. As illustrated in Table 2, 40% (4/10) of clinical studies published between 2010 and 2015 have used MSCs expanded in hPL or human serum (30–33), which provides evidence and confidence for a xeno-free era of MSC production. Second, in 90% (9/10) of recent clinical trials, MSCs have been generated from third-party donors (Table 2), and some patients received different batches of MSCs derived from two or more donors (15, 34, 35). This has further strengthened the concept response rates are independent on HLA-matching and reinforced the feasibility of using pre-manufactured “off-the-shelf” MSCs as a therapeutic agent (34, 35). On the other hand, recent clinical studies have also exposed significant limitations in the field. Although the reported response rates indicate some effect of MSCs on GvHD, their therapeutic efficacy remains ambiguous with complete and overall response rates varying from 10 to 65% and 61 to 91%, respectively, across the studies (Table 2). This can be attributed to multifactorial factors such as small patient cohort, lack of uniform efficacy measure and appropriate control groups in the analysis, heterogeneity in patient/MSC populations, and varying HSCT regimens. The lack of standardized protocols for MSC production and differences in dose/timing of MSC delivery could also contribute to the inconsistent results. These limitations highlight the need to interpret reported therapeutic efficacy with caution and preclude a definitive conclusion for the efficacy of MSCs in the treatment of GvHD.

Collectively, although the therapeutic efficacy of MSCs remains controversial, clinical studies consistently suggest that MSCs are safe to infuse in humans with no acute toxicity and no ectopic tissue formation, irrespective of their origin, culture conditions, and doses (17, 34, 39, 40). No association has been observed between MSC therapy and organ complications, death, or malignancy (41). This safety record allows future trials to be conducted using improved trial design and optimized practical procedures. Due to their immunosuppressive nature, whether MSC infusion could increase the risk of leukemia relapse and/or infectious diseases has been an area of concern. Results from clinical studies are highly controversial (40, 42, 43). This subject has been extensively discussed in a recent review (44). To date, MSC therapy in HSCT settings remains exploratory and experimental.

### Manufacturing of GMP-Compliant MSC Products

Among a spectrum of challenges, GMP-compliant cell production is one of the most critical steps. Translation of pre-clinical MSC amplification into clinical-grade large-scale expansion presents a big challenge for the development of a successful therapy. As with any cell therapy, the manufacturing process of MSCs for human use must follow GMP conditions and appropriate regulations to ensure product efficacy and safety. To achieve this, specialized GMP facilities, equipments, and trained staff are required. In addition, the unique characteristics of MSCs regarding cell source and cell culturing, including cell seeding, expansion, and culture medium, have to be considered. MSCs are mainly generated from BM, but umbilical cord and adipose tissue are also considered as well as a reliable source. Due to the low frequency of MSCs in BM (0.001–0.01%), large-scale *ex vivo* expansion is a pre-requisite to achieve the required cell dose of about 1–2 × 10^6^/kg, in total around 100–200 million cells/patient prior to clinical application (45). A very important factor to allow for a good expansion of MSCs is the density of plating the cells. As MSC are adherent cells, their growth is inhibited by reaching confluence. As a consequence, successive passaging of the cells has to be performed, and, typically after 3 weeks of culture, the proliferation rate and the differentiation potential declines. Furthermore, the increasing age of the donor is reported to be linked with a reduced expansion and multipotency (46). Details on standardization of the production of clinically applied products and further requirements have been summarized in several reviews (47, 48).

#### Development of Xeno-Free Expansion Medium

For the purpose of human applications, The International Society of Cellular Therapy (ISCT) recommends that reagents used for cell processing be free of xenogeneic products, due to the potential for infections, and that expansion be limited to early passages, due to the theoretical risk of cell senescence and malignant transformation. Conventionally, FCS is used for MSCs expansion for research applications and most clinical trials so far. FCS is a complex mixture of mitogenic factors which contribute to the maintenance and proliferation of MSCs *in vitro* (49). It is by nature ill-defined and exhibits batch-to-batch variability (50). It could be associated with the transmission of prions and undefined zoonosis as well as an increased risk of triggering adverse immune reactions resulting in the elimination of infused MSC, especially when multiple infusions are required (51). Therefore, the use of FCS is being criticized and strongly discouraged by the regulatory agencies, which urge for the development of GMP-compliant media, either serum- or animal-free, that can be standardized and used in both, research and clinical trials.

Over the last decade, numerous laboratories have been focused on the development of medium formulations that are either serum-free or use human blood-derived products, such as human autologous or pooled allogeneic serum, cord blood, and platelet derivatives (49, 51–55). Despite promising results with these culture supplements, the use of platelet derivatives, more specifically hPL has illustrated the best results. hPLs are manufactured by platelet disruption, using freeze/thaw protocols. Relatively standardized batches of hPL are produced by pooling platelet concentrates of several healthy donors (56). Repeated freezing/thawing of platelet concentrates allows the release of growth factors at a higher level that those in most FCS batches, such as basic fibroblast growth factor (bFGF), insulin growth factor 1 (IGF1), platelet-derived growth factor (PDGF), and transforming growth factor beta (TGF-β) (57). Several studies have demonstrated the use of hPL for MSC expansion provides increased proliferative capacity, while maintaining differentiation and immunomodulatory properties (57–59). These promising results have prompted the use of hPL-generated MSCs in clinical applications. Currently, 11 registered clinical studies are ongoing utilizing hPL-expanded MSCs for the treatment of GvHD, Crohn’s disease, and diabetes (www.clinicaltrials.gov; as for 10/2016). Meanwhile, GMP-grade complete media specially developed for MSC expansion are commercially available, which also achieve higher expansion rate and thereby shorten the production time and the associated risk of product contamination (48).

#### Culture Systems and Product Release

Classically, MSC expansion is performed in open culture systems using numerous plastic culture flasks or cell stacks. Manual handling steps for sequential cell passaging are labor intensive and time consuming, as well as bearing the risk for contamination. In this respect, automated and closed devices would simplify the manufacturing and increase product safety. Suitable bioreactors for MSC expansion on the market are the Quantum^®^ (Terumo) and Scinus Cell Expansion™ (Xpand Biotechnology). In addition, the CliniMACS Prodigy^®^ (Miltenyi Biotec) allows automated cell processing starting from sample preparation to cell culture and magnetic cell separation until the final formulation of the cellular product in a closed system by using single-use tubing sets (60). The instrument has the capability for preparation of mononuclear cells from BM samples using high-density gradient centrifugation prior to cell expansion. Additionally, magnetic enrichment steps for MSCs could be integrated into the manufacturing process, either before or after the expansion phase to further increase the purity of the cellular product.

Regarding the quality control for product release, the ISCT recommendation is to test for three characteristics of MSCs: (1) adherence to plastic; (2) expression of defined MSC cell surface markers, including positivity for CD73, CD90, and CD105 but negative for hematopoietic cell markers CD14, CD19, CD34, CD45; and (3) differentiation ability toward osteoblastic, chondrogenic, and adipocytic linages (61). Further tests, such as immunopotency assays and cytogenetic analysis remain at the discretion of the regulatory authorities (62). Ultimately, the most pressing issue relating to therapeutic efficacy is the fact that currently no standardized immune potency assay exists for quality control. This is partly complicated by their complex mechanism of action and the lack of understanding regarding MSC distribution and overall fate after infusion. However, a recent publication has described three tests defined in an ISCT workshop as potential release criteria: quantitative RNA analysis of selected gene products related to the cell’s immunomodulatory function, flow cytometry analysis of functionally relevant surface markers, and a protein-based assay of the MSC secretome (63). Together, these could provide appropriate guidance for releasing products, however not enough evidence currently exists to support their definitive use. Furthermore, a comprehensive understanding in the mechanisms of action of MSCs holds the key to successful development of future MSC therapies.

### MSC-Derived Extracellular Vesicles

Extracellular vesicles (EVs) are nanovesicles secreted by various cell types and are composed of a phospholipid bilayer, including transmembrane proteins and cell-specific receptors, enclosing cytoplasmic components. EVs are responsible for the horizontal transfer of bioactive proteins and genetic material, by internalization into endocytic compartments, fusion with plasma membranes, and/or by recognition of specific receptors (64). EVs can be easily isolated from cell culture medium and have been detected in a wide variety of bodily fluids (65–75). There are three major types of EVs: exosomes, microvesicles, and apoptotic bodies (76). A general description of these types and their corresponding characteristics can be found in Table 3. The two main types of EVs are microvesicles and exosomes, of which the latter are the most abundant.

**Table 3:** **Nomenclature and classification of the different types of vesicles**.

Characteristics	Exosomes	Microvesicles	Apoptotic bodies
Size	20–100 nm	50–1000 nm	500–5000 nm
Shape	Cup shaped	Irregular	Heterogeneous
Sedimentation	100,000 × *g*	Size dependent at 100,000 × *g*, 10,000 × *g*, and 2000 × *g*	Size dependent at 100,000 × *g*, 10,000 × *g*, and 2000 × *g*
Sucrose gradient	1.13–1.19 g/ml	1.04–1.07 g/ml	1.16 and 1.28 g/ml
Markers	Tetraspanins (CD63/CD9), Alix, TSG1, ESCRT components, flotilin	Integrins, tetraspanins, selectins, and CD40 ligand	Histones
Lipids	Cholesterol, sphingomyelin, ceramide, lipid rafts, phosphatidylserine	Phosphatidylserine	High amounts of phosphatidylserine
Origin	Endolysosomal pathway; intraluminal budding into multivesicular bodies and released by fusion of the multivesicular bodies with the cell membrane	Cell surface; outward budding of cell membrane	Cell surface; outward blebbing of apoptotic cell membrane
Contents	mRNA, microRNA, and other non-coding RNAs; cytoplasmic and membrane proteins (including HSP and cell-specific receptors)	mRNA, microRNA (miRNA), and other non-coding RNAs; cytoplasmic proteins and membrane proteins, including cell-specific receptors	Nuclear fractions and cell organelles

*ESCRT, endosomal sorting complexes required for transport; MVB, multivesicular bodies; HSP, heat-shock protein; mRNA, messenger RNA*.

*Table has been adapted from published literatures (90, 259, 260)*.

#### General Features of MSC-EVs

**Figure 1:**
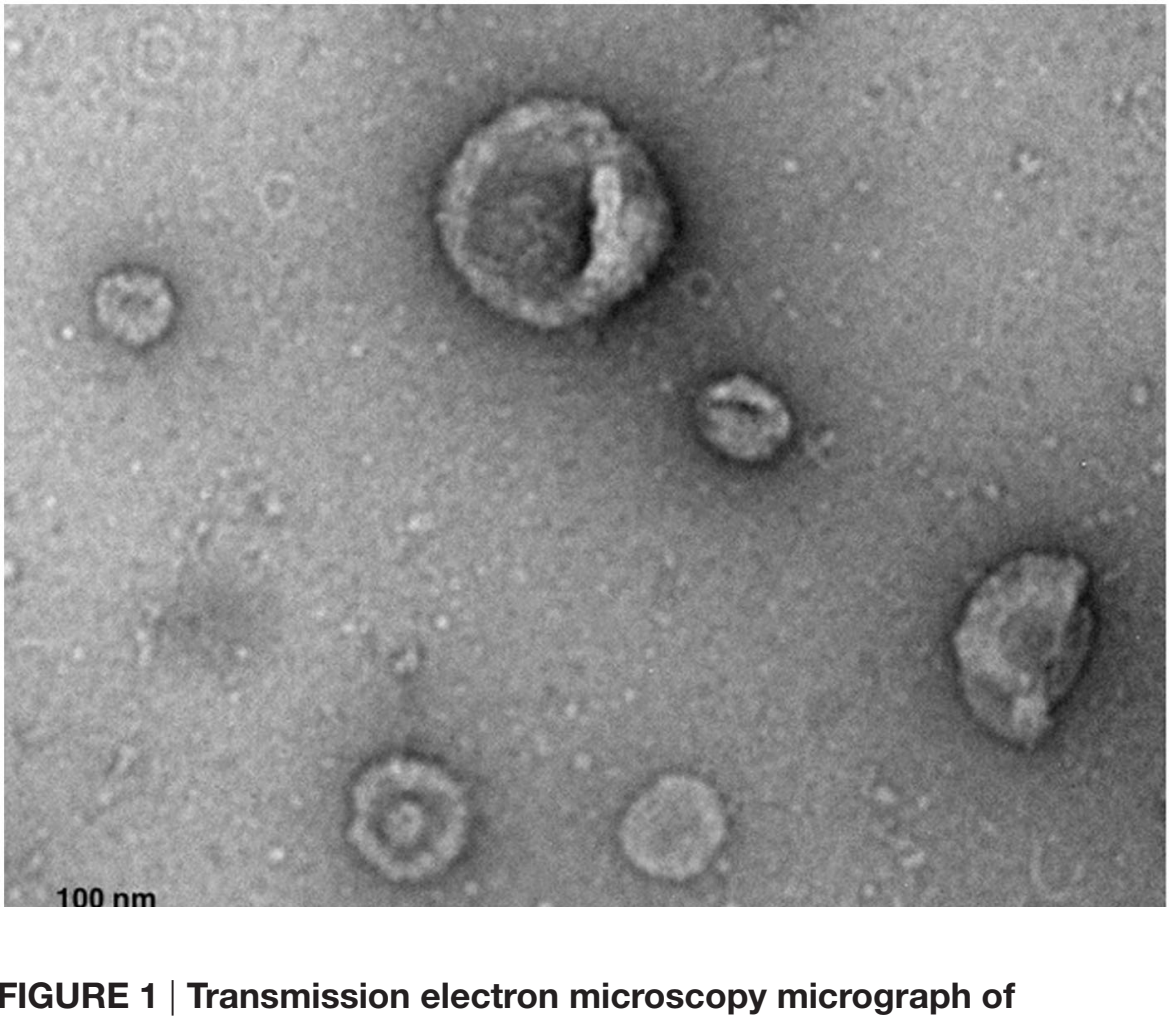
**Transmission electron microscopy micrograph of whole-mounted extracellular vesicles-purified human MSCs**. MSC-EVs exhibit a spheroid, cup-shaped morphology. Scale bar shows 100 nm. Photography courtesy of Monica Reis.

Mesenchymal stromal cells-extracellular vesicles are constitutively secreted by MSCs and can be identified by transmission electron microscopy as cup-shaped nanovesicles with sizes ranging from 20–150 nm in diameter (Figure 1). They are rich in adhesion molecules, such as intercellular adhesion molecule 1 (ICAM-1), lysosomal-associated membrane 2 (LAMP-2), tetraspanins (e.g., CD9, CD63, CD81), integrins (e.g., CD49C, CD49D), heat-shock proteins, cytoskeletal proteins, and membrane trafficking proteins, such as “Ras-related in brain” (67) and annexins (77, 78). Moreover, they express cell-specific molecules, including CD29, CD73, CD44, and CD105, and enclose proteins involved in MSC self-renewal and differentiation (GF, Wnt, TGF-β, MAPK, BMP, etc) (77). MSC-EVs also carry a variety of genetic material, including mRNA and non-coding RNA [pre-microRNA (miRNA), miRNA, tRNA, piRNA] (79–81). Significant importance has been given to MSC-EV shuttled miRNA, which has been shown to be functionally active and involved in the regulation of genes related to organ development, cell survival, and differentiation (82–85). The lipid composition of MSC-EV is still unknown; however, very recently, Lai and colleagues have reported an enrichment of phosphatidylserine (86). This lipid has been identified on the surface of various types of EVs, derived from various types of cells, and has been described as an evolutionary conserved immunosuppressive signal which promotes tolerance and prevents the activation of the immune system (87). Recently, Wei et al. have demonstrated that phosphatidylserine on the surface of MSC-derived microvesicles is essential for their uptake by human umbilical vein endothelial cells (HUVECs), however, the role of this lipid in MSC-EV-derived immunosuppression is still unexplored (88).

#### Common Procedures for EV Purification

Currently, differential ultracentrifugation represents the gold standard and most commonly used protocol for EV purification. This protocol involves several centrifugation steps at different speeds to eliminate cell debris and protein contaminants (89). EV sedimentation is usually accomplished by ultracentrifugation of the pre-cleared biofluid at speeds of 100,000 × *g*. This protocol varies across users which may lead to inconsistences in EV yields. In some protocols, EV sedimentation is accomplished at higher-speed ultracentrifugation (e.g., 140,000 × *g*) and longer centrifugations (e.g., 4–7 h). Alternatively, the last ultracentrifugation step can be replaced by microfiltration or followed by an extra purification step, e.g., sucrose-gradient centrifugation, which yields a cleaner EV population without co-precipitation or protein contaminants (89). Other EV purification methodology includes the use of commercially available kits based on polymer-precipitation and immune-capture using antibody-coated magnetic beads (90). The commercially available kits precipitate a wide range of vesicles, however, it may display concomitant precipitation of protein contaminants, while the immunolabeled beads only precipitates a restricted fraction of EVs and neglects others (90). Laboratories worldwide have been focused on the refinement of protocols to allow for a more robust purification and yield a purer EV population.

### Therapeutic Potential of MSC-Derived EVs

Since the initial identification of EVs in the conditioning medium of MSCs, increasing studies have demonstrated that MSC-EVs harness therapeutic effects. MSC-derived EVs have been shown to recapitulate the therapeutic effect of the parent cells in animal models of cardiac, kidney, and brain injuries and the observation MSCs have restricted migration and survival potential argues for the clinical use of EVs (91–94). The importance of MSC-EVs has also been identified as one of the mechanisms of MSC immunomodulation. MSC-EVs have been reported to modulate proliferation and differentiation of T cells, B cells, and monocytes (Table 4). Budoni et al. demonstrated that the effect of MSCs on B cell proliferation and differentiation could be fully reproduced by MSC-EVs and that this was inhibited in the presence of MSC-EVs in a CpG-stimulated peripheral blood mononuclear cell coculture system, in a dose-dependent manner (95). The effect of MSC-EVs on T cells was initially investigated by Mokarizadeh et al. in 2012. MSC-EVs were shown to express regulatory receptors, such as programed death ligand 1 (PD-L1), galectin-1, and membrane-bound TGF-β1, and were able to inhibit auto-reactive lymphocyte proliferation, promote the production of IL-10 and TGF-β, and increase apoptosis of recipient T cells (96). MSC-EVs seemed to induce tolerogenic signaling by prompting the generation of CD4^+^CD25^+^FoxP3^+^ Tregs (96). These findings were further corroborated by different independent studies which showed that MSC-EVs were capable of reducing proliferation and IFN-γ release of *in vitro* stimulated T cells in a dose-dependent manner and that one of the main mechanisms of MSC-EV to regulate T-cell proliferation and activation was the generation of *de novo* Tregs (97–99). Zhang et al. demonstrated that this effect was indirect and that MSC-EVs were preferentially taken up by splenocytes, which in turn polarized activated CD4^+^ T cells to that of a CD4^+^CD25^+^FoxP3^+^ Treg phenotype. In this study, the authors proposed that MSC-EVs are responsible for the activation of TLR-dependent signaling in macrophages, which leads to the induction of an IL-12^lo^IL-10^hi^ M2 phenotype. These M2 macrophages are then responsible for the generation of Tregs (100). Additionally, infusion of MSC-EVs led to enhanced survival of allogeneic skin grafts in mice (100). Recently, Favaro et al. demonstrated that MSC-EVs internalized by DCs impaired their *in vitro* maturation, with reduced expression of maturation markers CD86, CD80, and CD83, and an increase in IL-10 production by the EV-conditioned DCs (101).

**Table 4:** **Summary of the immunomodulatory potential of MSC-EVs**.

Target cells	Experimental approach	Source of EVs and isolation method	Results	Reference
PBMC	*In vitro* coculture	Human umbilical cord MSC	↓ Proliferation of CD8^+^ and CD4^+^	(99)
		UC (Sed.: 10,000 × *g*) and Exoquick	↑ Percentage of CD4^+^CD25^+^FoxP3^+^ Tregs	
			↑ TGF-β1 and IL-10; ↓ IFN-γ, IL-6, TNF-α	
Colon cells	TNBS-induced colitis model	Human BM-MSCs	↓ Pro-inflammatory cytokine levels in injured colons	(261)
		UC (Sed.: 100,000 × *g*)	Suppression of apoptosis	
			Inhibition of NF-kBp65 signal transduction pathways	
T lymphocytes	*In vitro* coculture	Human ASCs	Decreased T-cell activation and proliferation	(97)
		UC (Sed.: 100,000 × *g*)		
Auto-reactive lymphocytes	EAE mice	Murine BM-MSCs	EVs express PD-L1, galectin-1, and TGF-β1	(96)
		UC (Sed.: 100,000 × *g*)	Inhibition auto-reactive T-cell responses	
			↑ Apoptosis	
			↑ CD4^+^CD25^+^FoxP3^+^ Tregs	
PBMC from type I diabetes patients	*In vitro* coculture	Human BM-MSC	↓ IFN-γ production and ↑ TGF-β, IL-10, IL-6, and PGE2	(98)
		UC (Sed.: 100,000 × *g*)	↓ Level of Th17 cells and ↑ FoxP3^+^ Tregs	
B lymphocytes	*In vitro* coculture	Human BM-MSC	Inhibition of B-cell proliferation and differentiation	(95)
		UC (Sed.: 100,000 × *g*) and UF		
THP-1 MФ	*In vitro* coculture and *in vivo* injection of EVs in a mouse model of allogeneic skin grafting	Human ESC-MSC	↑ Anti-inflammatory cytokines	(100)
		HPLC	↓ Pro-inflammatory cytokines	
			TLR-dependent induction of M2-like phenotype	
			Treg cell expansion	
	*In vitro* coculture	LPS treated UC-MSC	MФ polarization *via* delivery of Let-7b by EVs and inhibition of TLR4 signaling pathway	(84)
		UC (Sed.: 100,000 × *g*)		
moDCs from type I diabetic patients	*In vitro* coculture	Human BM-MSC	EV-conditioned DCs exhibited immature phenotype	(101)
		UC (Sed.: 100,000 × *g*)	↑ IL-10, IL-6, and TGFβ	
			↓ IL-17 and Th17 cells	
			Treg expansion	

*Sed., sedimentation rate; UC, ultracentrifugation; UF, ultrafiltration; Tregs, regulatory T cells; EAE, experimental autoimmune encephalomyelitis; TNBS, 2,4,6 trinitrobenzene sulfonic acid; HPLC, high performance liquid chromatography; BM-MSC, bone marrow-derived MSC; ASC, adipocyte-derived stem cells; NF-kBp65, nuclear Factor kappa B p65; TGF-β1, transforming growth factor beta 1; IL-10, interleukin 10; IFN-γ, interferon gamma; IL-6, interleukin 6; TNF-α, tumor necrosis factor alpha; PD-L1, programed death ligand 1; PGE2, prostaglandin E2; TLR, toll-like receptor; IL-17, interleukin 17; Th, T-helper cell; MФ, macrophage; moDCs, monocyte-derived dendritic cells; LPS, lipopolysaccharide; FoxP3, forkhead box P3*.

Mesenchymal stromal cells-extracellular vesicles have also been tested in the context of HSCT and GvHD. A recent study has provided initial evidence that MSC-EV treatment combined with HSCs contributes to faster reconstitution of the hematopoietic microenvironment. In this study, MSC-EVs were shown to be enriched in miRNAs that promote UCB-CD34^+^ migration and engraftment in the BM niche (83). Amarnath et al. detected CD73-expressing EVs in MSC recipients in a mouse model of GvHD. These EVs were found to metabolize extracellular ATP into adenosine and, as a consequence, to inhibit Th1 cell effector function (102). In 2014, Kordelas et al. were the first to administer MSC-EVs in a steroid-refractory GvHD patient. MSC-EV preparations were shown to contain high concentrations of anti-inflammatory molecules IL-10, TGF-β, and HLA-G and were administered to the patient at intervals of 2 or 3 days for a period of 2 weeks. MSC-EV administration was well tolerated and no side effects were reported. The patient exhibited a 50% decrease in the production of the pro-inflammatory cytokines IL-1β, TNF-α, and IFN-γ and concomitant a reduction of diarrhea and cutaneous and mucosal GvHD, which remained stable for more than 4 months post MSC-EV treatment (103).

### Future Perspectives of MSC Therapy

#### Donor Source and the Use of Freeze–Thawed MSC Products

A long standing debate is the donor source of MSCs, particularly autologous versus allogeneic, and single-donor versus pooled donor batches (also called “master cell stocks”). Largely, the pros and cons of each relate to development costs and product safety. Autologous MSCs are innately safe from an immunological/infective perspective and obviate the search for a third-party donor. However, allogeneic MSCs would allow for product preparation in advance for infusion as an “off the shelf” treatment, without delays for the recipient. The advantages of master cell stocks are seemingly obvious, as they would allow mass production of MSCs for clinical use in multiple patients; as opposed to the need to isolate, expand, and quality check a batch of MSCs for every single recipient. However, not only would MSC production at an industrial scale prove costly, the potential for the contamination of multiple individuals with a single batch would require even more rigorous product analysis to ensure safety, which would only increase development costs further.

Another area of controversy is the clinical response and efficacy of using fresh (from culture) versus thawed MSCs. In earlier clinical trials, MSCs were infused into patients as thawed products, due to the benefit of cryopreservation allowing for long-term storage and use at a later date. However, recently the clinical effectiveness and safety of these products have been questioned (104). *In vitro*, it has been shown that post-thaw MSCs display a weaker immunomodulatory profile compared to their pre-freeze counterparts due to a heat-shock response, particularly in relation to weak IDO secretion (105). This seems to correlate with clinical outcomes, with reports of double the response rates in fresh compared to thawed MSCs for the treatment of HSCT complications (106). Despite aforementioned evidence, a recent study has examined the effect of cryopreservation on human MSC viability, immunomodulatory potency, and performance in an ischemia/reperfusion injury model. This study has observed that with modifications to standard cryopreservation methods over 95% MSC viability could be achieved upon thawing. These thawed high viability MSCs maintained their function in suppressing human mononuclear cell activation. Furthermore, the study has demonstrated that when viability is maintained, MSCs retained their therapeutic potency in an *in vivo* ischemia/reperfusion injury mode (107). This controversial evidence highlights potential risks as well as achievable hopes for an off-the-shelf therapy. Further studies are warranted to provide the field with a more definitive view.

From a safety perspective, concerns have also been raised regarding the possibility that post-thaw MSCs are associated with an increased rate of the so-called instant blood-mediated inflammatory reaction (IBMIR) (106). As seen with islets of Langerhans cells, this physiological process involves activation of a number of components, mainly the coagulation and complement cascades, leading to leukocyte and platelet activation, and subsequent tissue damage (108, 109). The extent of this, however, remains unclear, and more importantly, this has not been shown to have a negative impact on the safety profile of MSCs.

#### Mechanisms of Action of MSCs

**Figure 2:**
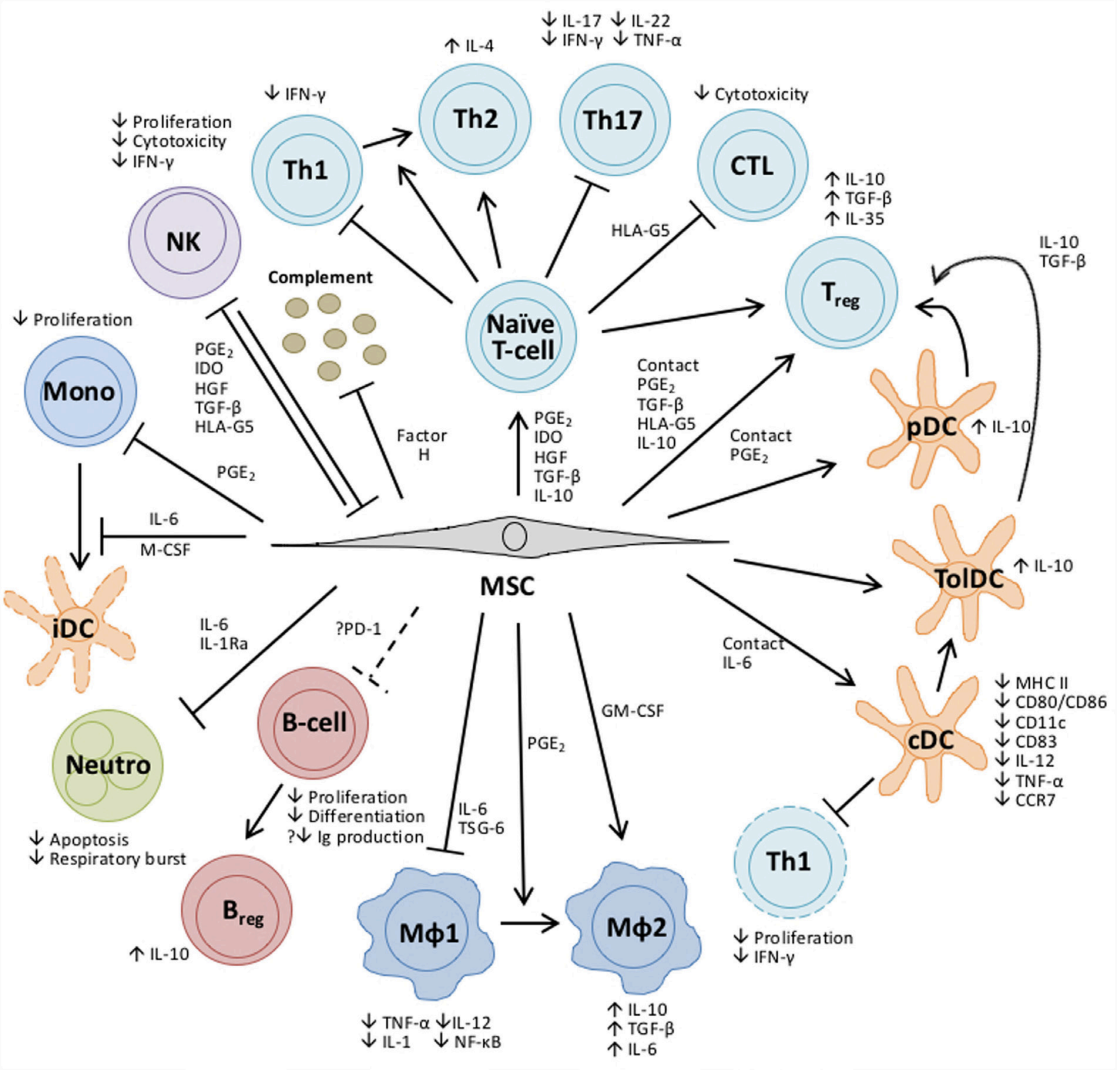
**Overview of the bioactive molecules secreted by MSCs and their impact on cells of the innate and adaptive immune response**. Some bioactive molecules are constitutively expressed by MSCs, while others are “licensed” by exposure to an inflammatory environment or upon TLR stimulation (241). Depending upon the bioactive secretion profile, MSCs can skew the differentiation of CD4^+^ T-helper cells into various T-cell subsets, each with distinct cytokine and gene expression profiles, can promote the generation of regulatory T cells (Tregs) and inhibit the proliferation of cytotoxic T cells (242–244). MSCs can modulate the development of conventional and plasmacytoid DC (245–247) while DCs generated in the presence of MSCs have functional properties typical of tolerogenic DCs (248–250). Similarly, MSCs can polarize macrophages of the classical M1 pro-inflammatory phenotype to that of an alternative anti-inflammatory M2 phenotype (215), or directly induce this alternative phenotype by coculture (251). In contrast to other cell types, MSC modulation of B-cell function is poorly understood and the findings are contentious. Results from *in vitro* experiments show that while MSCs impair the proliferation and terminal differentiation of B cells (252) they have also been shown to stimulate antibody secretion (253). More recently, data have emerged which suggests that MSCs can promote the induction of regulatory B cells (Breg) (254). Neutrophils are an important mediator of the innate response and MSCs have been shown to enhance their survival through an IL-6-mediated mechanism, concomitant with the downregulation of reactive oxygen species, thereby conserving the pool of neutrophils primed to respond rapidly to infection (255). MSCs inhibit the proliferation and differentiation of monocytes to immature dendritic cells (DCs) (245). Natural Killer (NK) cells and MSCs have a reciprocal relationship; MSCs can inhibit the proliferation and cytotoxicity of resting NK cells and their cytokine production *in vitro*, while activated NK cells can be cytotoxic to MSCs (256). MSCs constitutively secrete Factor H which inhibits complement activation (257), conversely the complement activation products C3a and C5a released upon tissue damage are chemotactic factors for MSCs (258), recruiting them to sites of injury. Abbreviations: CCR, C-C chemokine; CD, cluster of differentiation; cDC, conventional dendritic cell; CTL, cytotoxic T-lymphocyte; GM-CSF, granulocyte-macrophage colony-stimulating factor; HGF, hepatocyte growth factor; HLA, human leukocyte antigen; IDO, indoleamine 2,3-dioxygenase; IFNγ, interferon-γ; Ig, immunoglobulin; IL, interleukin; MФ, macrophage; MHC, major histocompatibility complex; Mono, monocyte; Neutro, neutrophil; NF-κB, nuclear factor kappa B; PD-1, programed cell death protein-1; pDC, plasmacytoid dendritic cell; PGE_2_, prostaglandin E_2_; TGFβ, transforming growth factor β; Th, T-helper cell; TNFα, tumor necrosis factor α; tolDC, tolerogenic dendritic cell; TSG, TNF-α-stimulated protein.

Despite their potential therapeutic benefits in GVHD treatment, the exact mechanisms of action of MSCs are yet to be fully elucidated. Increasing evidence has led to a common consensus that the efficacy of MSC therapy could be predominantly attributable to the release of soluble factors rather than long-term engraftment (110, 111). The MSC secretome includes an array of bioactive proteins, such as cytokines, growth factors, and chemokines. Their functions and interactions, together with relevant literatures, have been summarized in Figure 2. Ultimately, establishing a comprehensive understanding of how MSCs work holds the key to the development of successful MSC therapies.

#### Considerations in Using MSC-EVs for Therapies

Current research suggests MSC-EV-based therapy could potentially have significant clinical relevance. In comparison with MSCs, MSC-EVs are non-self-replicating hence no risk of aneuploidy, less likely to be modified by inflammatory environment and have a lower possibility of immune rejection owing to their small size and lower expression of membrane-bound molecules, including membrane histocompatibility molecules. MSC-EVs are also more stable than the parent cells, by virtue of their encapsulated cargo, EVs provide added protection to their contents from *in vivo* degradation, thus preventing problems associated with rapid breakdown of soluble molecules, such as cytokines, growth factors, and RNAs. In contrast to cell-based therapy, MSC-EV therapy can be easier to manufacture and safer, as they are devoid of cells and hence impose no danger of ectopic tissue formation. Additionally, they can be stored in non-toxic cryopreservatives at −20°C for 6 months with maintenance of biological activity (112). Despite these advantages, for clinical translation to be considered, it is essential to elucidate on the biological properties and the constituents of these vesicles, in terms of proteins and RNAs. MSC-EVs, as cellular products, are influenced by the secreting cells; therefore, it is inevitable that MSC heterogeneity will impact on EV cargo and biological effects. Distinct MSCs have been shown to display different abilities to produce cytokines and to respond to inflammatory licensing (113). Moreover, donor age and gender also affect the functional characteristics of MSCs (114). Current studies have not clarified the effect of inter-individual variability of MSC-EVs, and only a few studies have shown the effect of MSC licensing with inflammatory cytokines on the immunomodulatory potential to the EVs (84, 96). Furthermore, considerations regarding the immunomodulatory potency of the vesicles in relation to their cellular counterparts need to be taken into account. A recent report on the immunosuppressive effect of BM-MSCs and their derived EVs has shown the latter were considerably less potent in suppressing T cell proliferation and preventing B cell differentiation (115). EVs were also seen to be not as effective in modulating DC maturation as their parent cells (101). In the future, it will be important to investigate the effect of MSC variability and licensing on the molecular signature of their derived vesicles. This notwithstanding, data indicate MSC-EVs are capable, at least in part, of mediating immunomodulatory responses; however, further research is needed to unravel their mode of action, the development of standardized EV purification, characterization, and potency assays.

## Immunotherapy with Antiviral T Cells to Treat Infectious Complications

Opportunistic infections are serious complications affecting the morbidity and mortality of transplant patients (116). The most common infections in immunocompromised transplant recipients are caused by viral, bacterial, parasitic, and fungal pathogens (117). In immunocompetent individuals, the majority of these pathogens are controlled by the immune system, but in immunocompromised patients they can lead to prolonged recovery or hospitalization due to recurrent reactivations and can even influence the overall survival (116). The most important risk factors for post-HSCT infections are immunodeficiency and mucosal injury caused by conditioning regimen pre-transplantation (118), allogeneic transplantation with T cell depletion (119), delayed immune reconstitution due to immunosuppressive therapy for GvHD, and the pathogen serostatus of donor/recipients pairs (120). Taking the risk factors into account, preventive and pre-emptive treatments against these pathogens are important to promote engraftment, avoid relapse, and improve the overall survival. Bacterial, fungal, and parasitic infections can be treated with antibiotics, antifungals, or antiparasitic medications, but reconstitution of specific immunity is important. Latent virus reactivations or *de novo* infections can be treated with antiviral medications, but reactivation is only treated successfully or prevented by the recovery of anti-virus-specific T cells. The prophylaxis and the treatment of transplanted patients with traditional drugs might be effective by killing the pathogens or control replication; however, virus infections or invasive fungal infections (121) are often refractory to these treatments due to limited activity, drug resistance, or short-term drug protection (122, 123). Furthermore, antiviral and antifungal drugs have demonstrated significant toxicity, which raises a real concern for HSCT patients undergoing intensive drug treatments (124, 125).

Cytomegalovirus (CMV) is a latent herpes virus, which may lead to mild diseases at first contact or remains silent during most of the life of immune competent individuals. CMV is latently expressed in 30–60% of the population (126). CMV persists life-long in infected individuals in endothelial and epithelial cells, but is usually controlled by T cells specific for CMV (127). Thus, in immunocompetent individuals, the infection with CMV is not problematic, in immunocompromised individuals, like HSCT patients, it can trigger severe diseases. The most common manifestations of CMV disease are gastro-intestinal complications, pneumonia and interstitial pneumonitis, hepatitis, retinitis, and encephalitis (128). Furthermore, several studies have reported a correlation between CMV infection and reactivation with the onset or aggravation of GvHD, which makes the treatment of these patients even more difficult considering that the immunosuppression required for GvHD will increase CMV reactivation (129).

Epstein–Barr virus (EBV) is a herpes virus spread in more than 90% of the adult population with a life-long latency in B lymphocytes (130). EBV *de novo* infection or reactivation affects about 11 and 46%, respectively, of patients undergoing HSCT (131). The most life-threatening condition related to EBV infection in immunocompromised patients is the posttransplant lymphoproliferative disease (PTLD) (132).

Adenovirus (AdV) is a common latent virus, which presents at least 51 serotypes having various clinical manifestations, which make the decision for a therapeutic strategy more complicated than for other viruses. The infection occurs frequently during the childhood, but the most susceptible individuals are pediatric patients after HSCT (120). In these patients, AdV infection can cause hepatitis, pneumonia, encephalitis, myocarditis, gastroenteritis, or nephritis and when disseminated is associated with more than 50% of mortality risk (120, 133).

Cytomegalovirus, EBV, and AdV are the major viral pathogens involved in infection complications after HSCT. Other critical non-viral infections occurring in HSCT patients are invasive fungal infections mainly caused by fungal pathogens, such as *Aspergillus* and *Candida*. The mortality among posttransplant patients with IFI is between 1 and 13% and occurs in the majority of the cases within the first year after HSCT (134).

### Toward Adaptive T Cell Transfer for Treatment of Viral Infections

**Figure 3:**
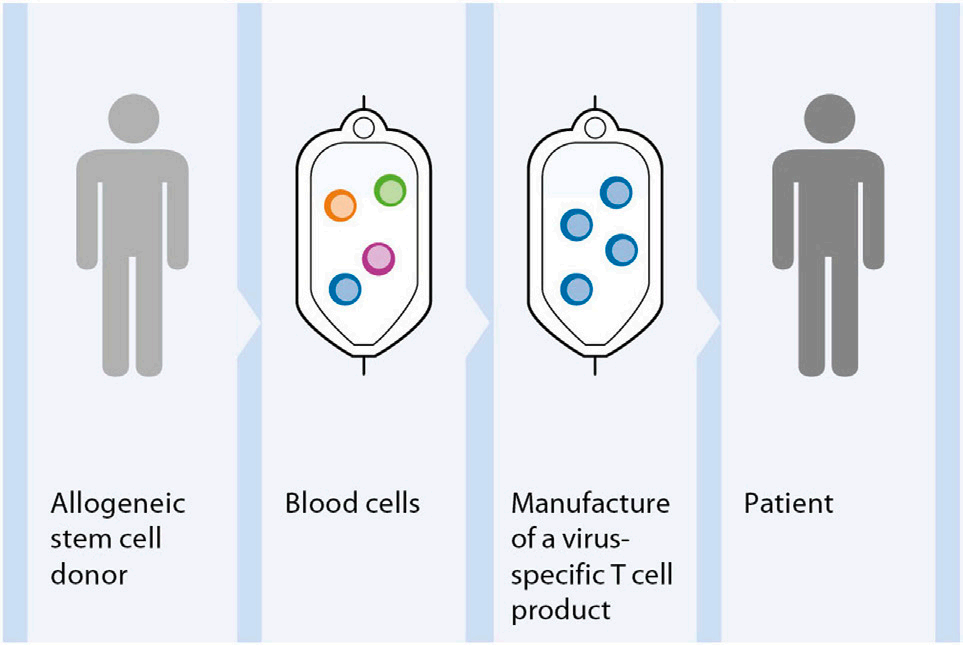
**Principle approach of adoptive T cell therapy for treatment of viral infections**. Out of peripheral blood of the HSCT donor the virus-specific T cells are selected. The generated T cell product is infused into the patient suffering of viral complications after allogenic HSCT.

Viral reactivation is the result of impaired function of the immune system, thus adoptive transfer of virus-specific T cells can help to restore virus-specific immunity after HSCT. Over the last 20 years, adoptive T cell therapy has become a potential alternative to pharmacologic treatments for patients with refractory posttransplant infections (135–138). Donor lymphocyte transfusion has been largely used in HSCT patients to prevent relapse by providing graft-versus-leukemia effect (GvL) although the development of GvHD has unfortunately been a concomitant risk (139, 140). In the early 1990s, it became evident that the practice of DLI was at the same time advantageous for the treatment of virus infections due to the presence of anti-virus reactive memory T cells among the lymphocytes from seropositive donors (141). Despite considerable benefits, the treatment of virus infections with DLI has demonstrated limitations concerning both safety and efficacy issues, due to the high presence of alloreactive T cells and to the low frequency of antigen-specific T cells (142, 143). These findings contributed to strategies increasing the number of antigen-specific T cells by selecting the donor target cytotoxic T cells and depleting the alloreactive T cells as an alternative immunotherapy for the reconstitution of the anti-pathogen immunity with a reduced risk of triggering GvHD (Figure 3). One of the pioneering studies published by Riddell et al. demonstrated the successful reconstitution of antiviral-specific T cell immunity in HSCT patients at high risk of developing CMV disease by the prophylactic transfusion of *in vitro* expanded CMV-specific CD8^+^ T cell clones (143). Although they could show the reconstitution of CMV-specific immunity, the expansion of virus-specific T cell clones had several drawbacks for integration into clinical practice. Since that time, innovative technological developments as well as novel basic immunological findings to improve and to disseminate the treatment of infectious diseases by adaptive anti-pathogen T cell transfer were developed.

### *In Vitro* GMP Manufacturing of Antiviral T Cell Products

**Figure 4:**
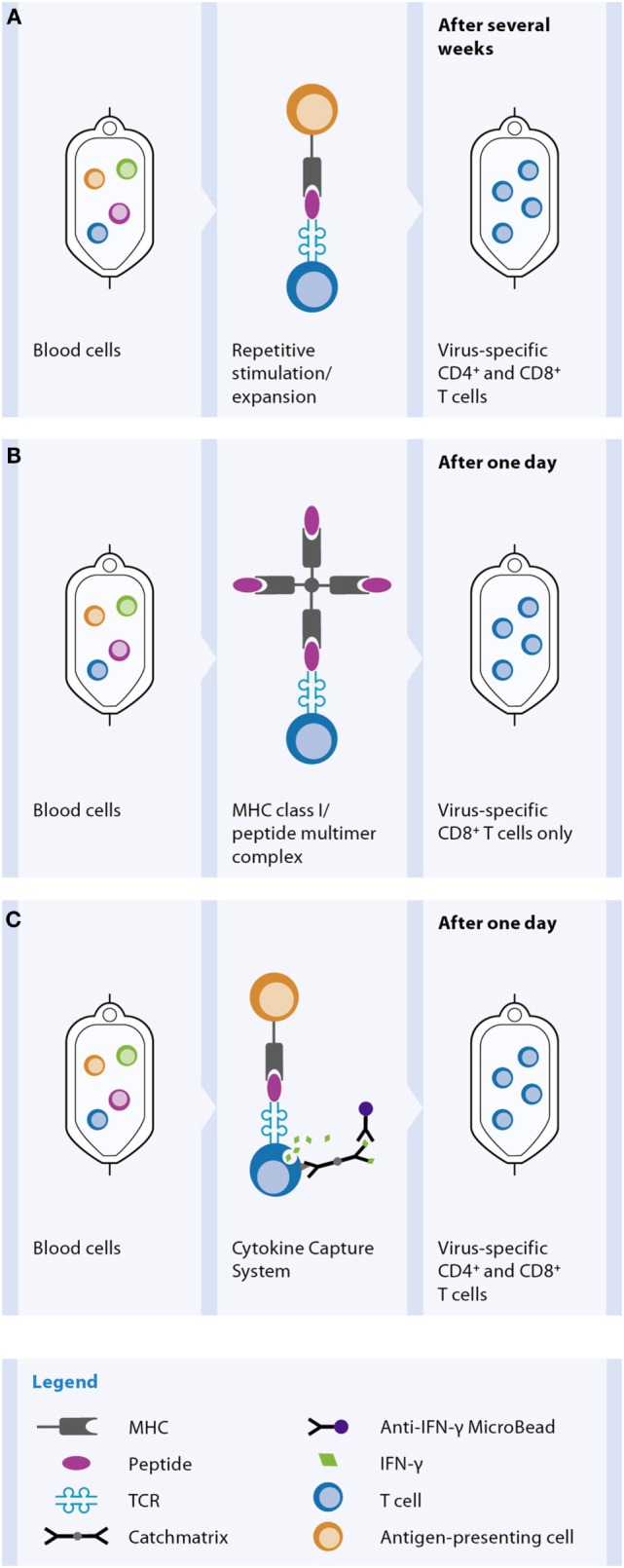
**Methods for *in vitro* generation of a virus-specific T cell product**. For the *in vitro* manufacture process blood is used as the cellular source, mostly derived from the stem cell donor. Selection of virus-specific T cell and thereby depletion of potentially alloreactive T cells from the blood can be achieved by different methods. **(A)** Activation and expansion: peripheral blood cells are incubated with viral antigen. Antigen-presenting cells (APC) phagocytose, process, and present the antigen as peptides on MHC molecules. Virus-specific T cells recognize their cognate viral antigenic peptide *via* the TCR, get activated, and later on start proliferating for several days. In many applications, additional repetitive antigen restimulations are performed to further increase the expansion and thereby the number and the purity of the virus-specific T cell population. Dependent on the viral antigen and APC used for the process, either CD4^+^ and/or CD8^+^ T cells are contained in the product. **(B)** MHC class I/peptide multimer technology: virus-specific T cells within peripheral blood become labeled by a MHC class I/peptide multimer reagent, which binds to the TCR of the viral peptide-specific T cells. After an additional labeling step with magnetic beads the CD8^+^ virus-specific T cells are magnetically enriched. **(C)** Cytokine-capture assay: peripheral blood cells are incubated with viral antigen, e.g., a peptide pool, for 4 h. APC present the peptides on MHC molecules to virus-specific T cells, which start producing IFN-γ. Cells are labeled with a catch matrix consisting of a CD45 antibody conjugated to an Anti-IFN-γ antibody. In this way, secreted IFN-γ is specifically captured on the cell surface of the activated virus-specific T cells. Subsequently, the cell-bound IFN-γ is detected with Anti-IFN-γ magnetic particles and the virus-specific T cells are magnetically enriched. Both CD4^+^ and CD8^+^ T cells are obtained by this method.

Basically two different strategies for depletion of potential alloreactive T cells and concomitant enrichment of relevant virus-specific T cells are established for the generation of GMP-grade antiviral T cell products (Figure 4). One strategy relies on conventional *in vitro* stimulation of blood cells with viral antigen and *in vitro* propagated antigen-presenting cells (APC), like EBV-transformed B cells, and repetitive restimulation and long-term culture to gain T cell clones or lines (143, 144) (Figure 4A). Despite successful optimization and simplification of multiple steps within this manufacturing over the last year to yield clinically practical protocols resulting in effective and safe T cell lines, a main disadvantage of these cell products is the long and laborious preparation time of at least 10 days (145). The development of new magnetic selection methods to obtain the rare virus-specific T cells out of peripheral blood, based on either IFN-γ secretion [CliniMACS^®^ Cytokine Capture System (CCS) (IFN-gamma)] enables or peptide/MHC multimer labeling, allowed significant reduction of the preparation time of the cellular product under GMP conditions from one to two working days.

The peptide/MHC multimer technology allows selection of peptide-specific CD8^+^ T cells out of a blood sample according to the magnetic labeling of the antigen-specific T cell receptor (TCR), without the need of prior *in vitro* stimulation step (Figure 4B). The CliniMACS^®^ CCS (IFN-gamma) requires stimulation of peripheral blood samples with antigen like peptides or proteins for about 4–16 h to induce IFN-γ production by the virus-specific T cells (Figure 4C). The secreted IFN-γ is specifically caught onto the cell surface of antigen-activated T cells using a capture matrix. The subsequent recognition of IFN-γ-secreting cells with magnetic beads conjugated to anti-IFN-γ antibodies enables its enrichment.

Both methods yield rapid and effective production of antigen-specific T cells. The advantage of the CCS over peptide/MHC multimer technology is a parallel stimulation and selection of antigen-reactive CD4^+^ as well as CD8^+^ T cells. Although CD8^+^ cytotoxic T lymphocytes (CTLs) are responsible for the fast antiviral response, it has been shown that the presence and help of antigen-specific CD4^+^ T cells is essential to activate the CTL and maintain long-term immunity (146). Moreover, the CCS enables generation of a cell product consisting of multiple viral epitopes of either one or more antigenic viral proteins. Whereas the number of available peptide/MHC multimer reagents is limited to the most common HLA/epitope specificities, the cytokine-capture assay is suitable for isolation of specific T cells independent of HLA allotypes. A disadvantage of the IFN-γ secretion assay technology compared to peptide/MHC multimer technology is the need for a short-term (4 h) incubation phase for antigenic stimulation. However, exactly this technological feature makes it possible to generate tailored T cell products for patients by choosing on the required viral antigen, either peptides, pools of peptides, proteins, and even use of multiple antigens. Meanwhile a whole panel of viral protein antigens is available as pre-pooled GMP-grade peptide cocktails, covering CD4^+^ as well as CD8^+^ T cell epitopes without HLA restriction.

Despite the possibilities of adaptive virus-specific T cell therapy the number of clinical sites, which have GMP manufacturing unit and processes and thus offering such a treatment option to patients, is limited. One of the general obstacles of cell therapy is the complexity of the clinical manufacturing. Beside the demands on the infrastructure with clean rooms and various instruments, the generation of antigen-specific T cell products requires several different handling and intervention steps during the production process and skilled and well-trained operators are needed. To guarantee robust and reliable processes as well as safe and effective clinical products, a standardization of the cell manufacturing is essential, which can be accomplished by automation. A newly developed cell processing device, the CliniMACS Prodigy^®^, enables to run the complete CCS in an automated and closed system (60, 147). The cells are processed from the first until the last step within a closed and single-use tubing set. All process steps, i.e., cell preparation, cell stimulation, labeling and washing steps, magnetic enrichment, and final formulation are performed automatically. Only a minimum of operator action is necessary to set-up sterile connections of all starting materials (blood sample, antigen, buffers, cell culture media, labeling, and separation reagents) to the tubing set, for programing the desired time of the process end, and for cell sampling to allow their quality control.

### Quality of the Cellular Products Determines Clinical Outcome

#### Clinical Benefits Are Detected upon Transfer of Low Numbers of T Cells

The cell numbers obtained with either system for rapid magnetic *ex vivo* selection of virus-specific T cells is limited due to the low frequency of virus-specific T cells within peripheral blood. *In vitro* expansion of the specific T cells was considered to be essential for a successful adoptive therapy as in early clinical studies the number of transferred T cells were as high as several million cell/m^2^ body surface area (148, 149). However, various investigators treated patients with CMV-, EBV-, or AdV-specific T cells directly obtained after *ex vivo* isolation using the CCS and reported clinical efficacy (138, 150–153). Thus, this low number of transferred cells most probably are compensated by their high *in vivo* proliferating capacity in the lymphodepleted host, thus leading to sufficient antigen-specific T cell immunity and successful treatment of viral infections. It has been shown for tumor-infiltrating T cell products that longer periods of *in vitro* expansion reduce the clinical efficacy *in vivo*, hypothesized to be the result of enhanced terminal differentiation of cells (154). The number of virus-specific T cells that can be isolated *ex vivo* using either method depends mainly on the frequency of specific T cells in donors’ peripheral blood. Usually the number of enriched T cells allows the transfer of less than 1 × 10^5^/kg body weight of the patient, i.e., the number of transfused T cells is below the number of unselected T cells regarded as critical for GvHD induction (MUD/MRD: 1 × 10^6^/kg body weight). Thus, methods for rapid generation of cellular antiviral T cell products are of advantage compared to long-term cell culturing processes.

#### Cotransfer of CD4^+^ T Cells Support *In Vivo* Effector Function of CD8^+^ T Cells

Controversial data on the protective role of CD4^+^ and CD8^+^ T cells, the benefit of transferring both, antigen-specific CD4^+^ and CD8^+^ T cell subsets, or CD8^+^ T-cells alone still exist and need to be discussed. The prophylactic infusion of CMV-specific CD4^+^ T cells in patients without CMV-specific T helper response has been shown to increase the frequency of CMV-specific T cells in both CD4^+^ and CD8^+^ T cell subpopulations and to eradicate the virus successfully (149). On the other hand, the transfusion of CMV-specific CD8^+^ T cells has been likewise efficient in clearing the viremia and increasing the frequency of donor CMV-specific CD8^+^ T cells as well as recruiting CD4^+^ T cells in the recipients (155). Riddell et al. and Walter et al. have transfused CMV-specific T cell clones and reported a progressive decrease of transferred CMV CD8^+^ T cell clones in patients who lacked CD4^+^ T cells (143, 148). Since then, several other studies have demonstrated the critical role of CD4^+^ T cells in both maintaining the functionality of cytotoxic CD8^+^ T cells (156, 157) and directly fighting the viral infection (158). Furthermore, in a multicentre study, Leen et al. observed that transfusion of either CD4^+^ or CD8^+^ T cells were equally protective against viral infections (159). The CD4^+^ T cell population remains, however, a controversial issue for adoptive immunotherapy, since several studies have reported a higher alloreactive potential of this T cell subset (160, 161).

#### Reduced Alloreactivity in *In Vitro*-Generated T Cell Products

The allogeneic reactivity of pathogen-specific T cells has been largely investigated and their potential to elicit GvHD needs still to be clarified, particularly with HLA-mismatched donors. Several *in vitro* studies have reported the cross-reactive potential of expanded virus-specific T cells toward allogeneic-HLA antigens (162–166). Single-viral antigen CD4^+^ and CD8^+^ T cell lines or clones, specific for CMV, EBV, VZV, and influenza virus, have shown *in vitro* to recognize and lyse allo-HLA class I and class II molecules also expressed on normal cell subsets (164). Long-time culture and the generation of clones under repeated immune stimulation may contribute to the *in vitro* alloreactivity of T cell clones, reported. In the clinical setting, this alloreactivity has not been reported, not even in HLA-mismatched clinical conditions (163).

**Figure 5:**
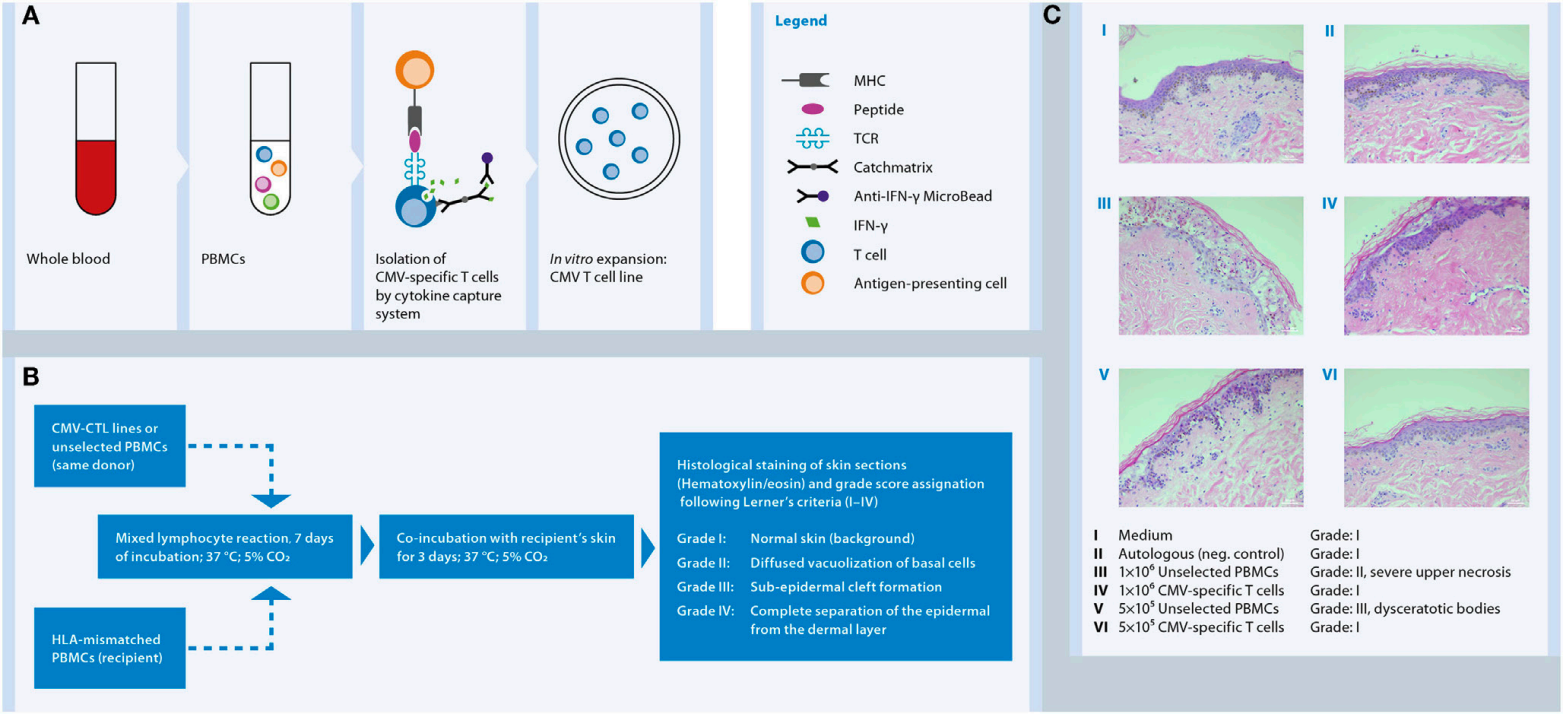
***In vitro* human skin explant assay as a model to investigate the potential of third-party CMV-specific T cells to elicit GvHR in an HLA-mismatched system**. **(A)** CMV-specific T cells were isolated from blood of seropositive donors by IFN-γ secretion assay and expanded *in vitro* between 2 and 4 weeks with IL-2 and irradiated feeder cells. **(B)** CMV-specific T cell lines and unselected PBMCs from the same donor where exposed to HLA-mismatched PBMCs (recipient’s cells) in a mixed lymphocyte reaction for 7 days followed by incubation with recipient’s skin for further 3 days. Then skin biopsies were collected, fixed in formalin, and stained with hematoxylin and eosin. **(C)** The histopathological damage in the skin biopsies displays a readout of the allogeneic-HLA reactions caused by T cell activation. The images show that CMV-specific T cells do not cause GvHR (Grade I) as opposed to Unselected PBMCs (Grade II and III) from the same donor.

*In vitro* data clearly showed a high degree reduction of alloreactivity by selection and expansion of CMV- and AdV-specific T cells using the CliniMACS^®^ CCS (IFN-gamma) is achieved (167–169). One limitation of the data above is that the tests were not performed versus the recipients’ material. In practice, alloreactivity testing of the donor material versus the recipient material is not feasible due to the time it takes and the necessary collection of the relevant tissue since GvHD can affect the skin, the gut, and the liver. Moreover, there was no alloactivation reported in AdV-specific T cells stimulated with third-party HLA-matched unrelated donor cells in a mixed lymphocyte reaction (MLR) setting when compared with autologous stimulation, but a residual 28% of alloreactivity was shown in the HLA-mismatched MLR setting (150). Very recently, our team has demonstrated that CMV-CTL isolated by IFN-γ secretion assay and further *in vitro* expansion did not induce relevant cutaneous GvH tissue damage in the *in vitro* skin explant model while maintaining high level of antiviral activity (170). At low cell doses (5 × 10^5^) none of CMV-CTLs led to GvH reactions in the HLA-mismatched recipient’s skin, whereas at the high cell dose (1 × 10^6^) two of nine CMV-CTLs induced a mild GvH skin damage (Figure 5). Our observations contribute to further elucidate the knowledge on the immunogenicity of antiviral T cells supporting simultaneously their safety use in the clinical practice.

Furthermore, it is important to correlate the phenotype and functionality of the infused cells with the clinical outcome.

### Clinical Trials Using *Ex Vivo* Magnetically Enriched Virus-Specific T Cells

Clinical trials to date have confirmed safety and efficacy of the adoptive transfer of virus-specific T cells. The Tables 5–7 summarize the data of clinical studies performed with donor-derived CMV-, EBV-, and AdV-specific T cell products, either using T cell lines or directly *ex vivo* isolated T cells, administered for therapeutic or pre-emptive treatment after HSCT. We are going to discuss in more detail below the virus clearance and kinetics of virus-specific immune recovery after application of cellular therapies based on the two methods for *ex vivo* isolation of virus-specific T cells, namely the IFN-γ secretion assay and the peptide/MHC multimer selection technologies.

**Table 5:** **Clinical trials with therapeutic treatment of CMV-specific T cells**.

Reference	Method	No. pts	Results	Dose
Einsele et al. (149)	*In vitro* stimulation and expansion of CMV-specific polyclonal CD4^+^ and CD8^+^ T cells	8	5/7 evaluable pts eliminated infection	10^7^ cells/m^2^
Peggs et al. (262)	*In vitro* stimulation and expansion of CMV-specific polyclonal CD4^+^ and CD8^+^ T cells	16	Pre-emptive therapy: 8/16 did not require antiviral treatment	0.2–1 × 10^5^ T cells/kg
Bao et al. (263)	*In vitro* stimulation and expansion of CMV-specific polyclonal CD4^+^ and CD8^+^ T cells	7	3/7 pts cleared infection 1/7 pts reduced viral load	2.5–5 × 10^5^ CMV-specific CD3^+^ cells/kg
Blyth et al. (264)	*In vitro* stimulation and expansion of CMV-specific polyclonal CD4^+^ and CD8^+^ T cells	21	Pre-emptive therapy: 13/21 did not require antiviral treatment	2 × 10^7^ CMV CTLs/m^2^
Koehne et al. (265)	*In vitro* stimulation and expansion of CMV-specific polyclonal CD4^+^ and/or CD8^+^ T cells	16	14/16 pts eliminated infection	5 × 10^5^–3 doses with 1 × 10^6^ T cells/kg
**Total**	***In vitro* stimulation and expansion**	**68**	**23/30 responders (w/o pre-emptive therapy)**	
Feuchtinger et al. (171)	Direct isolation of CMV-specific CD8^+^ and CD4^+^ T cells using the CCS	18	15/18 responders	1.2–166 × 10^3^ cells/kg
Peggs et al. (137)	Direct isolation of CMV-specific CD8^+^ and CD4^+^ T cells using the CCS	11	Pre-emptive therapy: 2/11 did not require antiviral treatment	10^4^ CD3^+^ T cells/kg
Meij et al. (266)	Direct isolation of CMV-specific CD8^+^ and CD4^+^ T cells using the CCS	6	6/6 patients eliminated infection	0.9 × 10^4^–3.1 × 10^5^ cells/kg
**Total**	**Direct isolation using the CCS**	**35**	**21/24 responders (w/o pre-emptive therapy)**	
Cobbold et al. (267)	Direct isolation of CMV-specific CD8^+^ T cells using MHC-I-tetramers	9	8/9 patients eliminated infection	1.2–33 × 10^3^ cells/kg
Schmitt et al. (155)	Direct isolation of CMV-specific CD8^+^ T cells using MHC-I-streptamers	2	Control of CMV-viremia in both patients	0.37 and 2.2 × 10^5^ cells/kg
Uhlin et al. (268)	Direct isolation of CMV-specific CD8^+^ T-cells using MHC-I-pentamers	5	4/5 responders	0.8–24.6 × 10^4^ cells/kg
**Total**	**Direct isolation using MHC-I-multimers**	**16**	**14/16 responders**	

**Table 6:** **Clinical trials with therapeutic treatment of EBV-specific T cells**.

Reference	Method	No. pts	Results	Dose
Rooney et al. (144)	*In vitro* stimulation and expansion of EBV-specific CD8^+^ T cells	10	Therapy: 3/3 responders Prophylaxis: 7/7 virus free	0.2–1.2 × 10^8^ cells/m^2^
Haque et al. (269)	*In vitro* stimulation and expansion of EBV-specific CD8^+^ and CD4^+^ T cells	8	4/8 Remission	10^6^ cells/kg
Haque et al. (270)	*In vitro* stimulation and expansion of EBV-specific CD8^+^ and CD4^+^ T cells	33	14/33 complete remission 3/33 partial response	2 × 10^6^ cells/kg
Heslop et al. (271)	*In vitro* stimulation and expansion of EBV-specific CD8^+^ T cells	114	Therapy: 11/13 complete response Prophylaxis: All PTLD free	1–5 × 10^7^ cells/m^2^
Doubrovina et al. (272)	DLI or *in vitro* stimulation and expansion of EBV-specific CD8^+^ T cells	19	13/19 complete response	10^6^ cells/kg
Gallot et al. (273)	*In vitro* stimulation and expansion of EBV-specific CD8^+^ and CD4^+^ T cells	11	4/10 responders	5 × 10^6^ cells/kg
**Total**	***In vitro* stimulation and expansion**		**52/86 responders (w/o prophylaxis)**	
Moosman et al. (152)	Direct isolation of EBV-specific CD8^+^ and CD4^+^ T cells using the CCS	6	3/6 responders	0.4–9.7 × 10^4^ cells/kg
Icheva et al. (151)	Direct isolation of EBV-specific CD8^+^ and CD4^+^ T cells using the CCS	10	7/10 responders	0.15–53.8 × 10^3^ cells/kg
**Total**	**Direct isolation using the CCS**	**16**	**10/16 responders**	
Uhlin et al. (268)	Direct isolation of EBV-specific CD8^+^ T cells using MHC-I-pentamers	1	1/1 complete response	1.8 × 10^4^ cells/kg
**Total**	**Direct isolation using MHC-I-multimers**	**1**	**1/1 responder**	

**Table 7:** **Clinical trials with therapeutic treatment of AdV-specific T cells**.

Reference	Method	No. pts	Results	Dose
Geyeregger et al. (274)	*In vitro* stimulation and expansion of AdV-specific CD8^+^ and CD4^+^ T cells	2	1/2 complete response	10^4^ CD3^+^ cells/kg
			1/2 partial response	
**Total**	***In vitro* stimulation and expansion**	**2**	**2/2 responders**	
Feuchtinger et al. (150)	Direct isolation of AdV-specific CD8^+^ and CD4^+^ T cells using the CCS	9	4/9 responders	1.2–50 × 10^3^ cells/kg
Qasim et al. (153)	Direct isolation of AdV-specific CD8^+^ and CD4^+^ T cells using the CCS	5	3/5 responders (cleared adenoviremia)	10^4^ cells/kg
Feucht et al. (138)	Direct isolation of AdV-specific CD8^+^ and CD4^+^ T cells using the CCS	30	21/30 responders	0.3–24 × 10^3^ CD3^+^ cells/kg
**Total**	**Direct isolation using the CCS**	**44**	**28/44 responders**	
Uhlin et al. (268)	Direct isolation of AdV-specific CD8^+^ T cells using MHC-I-pentamers	1	No response	3.1 × 10^4^ and 1.7 × 10^4^ cells/kg
**Total**	**Direct isolation using MHC-I-multimers**	**1**	**0/1 responder**	

#### Clinical Trials Using CliniMACS^®^ Cytokine Capture System (IFN-Gamma)

Several studies used the IFN-γ secretion assay to select antigen-specific T cells (Tables 5–7). Feuchtinger and colleagues published the clinical experience on 13 patients treated with the infusion of pp65-specific IFN-γ-secreting CD4^+^ and CD8^+^ cells for refractory CMV infections or CMV disease after HSCT (171). It was observed that *in vivo* expansion of transferred cells was correlated with clearance or significant reduction of viremia. Furthermore, expansion was seen in CD4^+^ and CD8^+^ T cells and cells could be detected *in vivo* within an average of 3–6 weeks. The transferred pp65-specific T cell immunity could be detected for more than 6 months after infusion in single patients (171). Moosmann and colleagues used the IFN-γ capture assay and stimulation with peptides derived from EBV antigens to generate EBV-specific T cells to treat PTLD induced by EBV (152). Three out of six patients had complete and stable remission after failing treatment with rituximab, an anti-CD20 antibody together with low numbers of CD4^+^ and CD8^+^ EBV-specific T cells. Non-responders suffered from the late-stage disease with multiorgan dysfunction at the time of T cell transfer. In two responders, long-term follow up was possible, showing that EBV-specific T cells rapidly expanded upon transfer, high levels were maintained for approximately 6 months then the numbers declined, according to the characteristic expansion and contraction of antigen-specific T cells, and stabilized at levels characteristic for healthy individuals, providing protection for at least 2 years after transfer. Detailed analyses of cell differentiation markers early after transfer showed that EBV-specific CD8^+^ T cells had an effector memory phenotype (CCR7^−^ CD45RA^−^), which after contraction evolved into central memory (CCR7^+^ CD45RA^−^) and terminally differentiated effector cells (CCR7^−^ CD45RA^+^). It has also been shown that infusion of AdV-specific IFN-γ^+^ T cells was successful and their expansion *in vivo* correlated with decreased viral load (138, 150). The analysis of four AdV-specific T cell products before treatment revealed that the majority of cells were of effector memory phenotype, identified based on the expression profiles CCR7^−^ CD45RA^−^ and CD62L^−^ CD45RO^+^, and a minority of central memory phenotype, characterized as CCR7^+^ CD45RA^−^ and CD62L^+^ CD45RO^+^ (138). Further investigation on tracking of the infused cells and correlating the phenotype and functionality of the infused cells with the clinical outcome will in addition help to define the optimal conditions for a successful and long-lasting effect of the adoptive transfer.

#### Clinical Trials Using the Peptide/MHC Multimer-Based Selection

Nowadays, this technique is used for adoptive transfer, since it has been shown that antigen-specific CD8^+^ T-cells selected with peptide/MHC multimers induced long-lasting immune responses without increasing the risk for GvHD (Tables 5–7). The development of “reversible” TCR staining with streptamers allowed selection of the phenotypically and functionally unchanged cells (172, 173). Schmitt and colleagues reported results from the study on two patients treated with CMVpp65-specific T cells for recurrent CMV antigenaemia after HSCT (155). For one of the donors the phenotype and function of cells after transfusion was analyzed. Donor-derived CMV-specific T cells from the cellular product rapidly expanded *in vivo*, showed early after transfusion an effector memory phenotype (CCR7^−^ CD45RA^−^), acquired effector phenotype (CCR7^−^ CD45RA^+^) at later timepoints, and were capable of secreting IFN-γ upon *in vitro* stimulation. In both patients, clearance of the CMV reactivation without any signs of GvHD was observed. Additionally, Odendahl and colleagues showed in a pre-clinical study the potential of clinical-scale CMV streptamer-selected T cells. In this study, 22 cell products displayed excellent viability, cytotoxicty, and purity with effectively removed selection reagents (174). Recently, a GMP-compliant protocol using the streptamer technology was implemented to enrich EBV- and AdV-specific T cells. Because of the very low frequencies of EBV- and AdV-specific T cells in the starting material, the purity (among CD3^+^ cells) of the large-scale cell product was poor, up to 44 and 6.7%, respectively. However, an increase in purity was achieved by small-scale selection or simultaneous application of EBV- and AdV-streptamers. An IFN-γ response was seen in most of the products and cells were predominantly of the effector memory (CD62L^−^ CD45RA^−^) or central memory phenotype (CD62L^+^ CD45RA^−^), thus those cells are suitable for clinical use (175).

### Future Perspectives

#### Generation of Multipathogen-Specific T Cells

Adoptive transfer of multi-antigen-specific T cells is a promising approach in restoring antigen-specific immunity and preventing or treating infectious complications after HSCT. Several strategies have been developed to simultaneously select T cells specific for viral and/or fungal pathogens. Initial studies focused on CMV, EBV, and AdV, using a clinical-grade AdV vector Ad5f35 with expression of the CMV antigen pp65 transgene, which permitted transduction of APC like DCs or EBV-transformed B cells to successfully stimulate and expand virus-specific T cells (164, 176–178). A new enrichment strategy based on the activation-dependent CD154 (CD40L)-expression (transient expression on activated CD4^+^ and to lesser extent on activated CD8^+^ T cells) and subsequent expansion of T cell has been introduced to production of multi-pathogen-specific T cells without the need to genetically modify APC. This technique allowed generation of alloantigen-depleted CD4^+^ and CD8^+^ T cell lines within 14 days with high specificity for the most common posttransplantation pathogens. These T cell lines showed extensive proliferative capacity and confirmed functionality *in vitro* (179). Recently, the use of either DNA plasmids or peptide pools to pulse APC has been validated to avoid safety and regulatory issues associated with transduction of APC using viral vectors. The combination of the peptide mixture approach or transfection of DC with plasmids with expansion in gas permeable rapid expansion (G-Rex) bioreactors provided further advances, increasing both feasibility and applicability of T cell therapy (180). These rapidly (10–12 days) expanded multi-virus-specific T cells provided effective antiviral protection in clinical trials (121, 159). Certainly, the short-term activation concomitantly with peptide pools from multiple viral antigens in combination with the CliniMACS^®^ CCS (IFN-gamma) provides the most simplest and fastest way for simultaneous GMP-grade selection of CMV-, EBV-, and AdV-specific CD4^+^ and CD8^+^ T cells.

#### Broadening the Clinical Use of Adoptive T Cell Therapy

Several barriers prevent the broader use of virus-specific T cell therapies after HSCT. One of the main hurdles is associated with the complexity of GMP-grade cell manufacturing. More details and suitable solutions are described for generation of virus-specific T cells in Section “*In Vitro* GMP Manufacturing of antiviral T Cell Products” and of gene-modified T cells in Section “Complexity of the Cell Manufacturing Process” of this article. A second main problem is connected with pathogen-naïve donors and umbilical cord blood transplants. For immunotherapy with cells derived from pathogen-naïve donors or cord blood, *in vitro* priming of the donor T cells with APC pulsed with antigen or genetically modified APC can be introduced (177). Another option is the transfer of virus-specific TCR genes into donor primary T cells by viral vectors (181). The antigen-specific responses in recipient can be boosted also by the vaccination with peptide-loaded donor-derived DC (182). Apart from above mentioned strategies, the selection of the virus-specific T cells from healthy seropositive third-party donors is an attractive alternative. Haque and colleagues showed for the first time that partially matched third-party EBV-CTL led to the control of PTLD after solid organ transplantation (183). Also post-HSCT successful treatments of refractory viral infections (CMV, EBV, AdV) with third-party virus-specific T cells were reported (177, 184). A detailed summary on clinical results of third-party-derived virus-specific T cell administration is found in a recent review written by O’Reilly and colleagues (185). The first promising results using virus-specific T cells from third-party donors initiated the idea of donor registries and biobanks with the cryopreserved antigen-specific T cells, which could provide “off the shelf” immunotherapy product (185).

The introduction of rapid manufacturing technologies such as magnetic enrichment processes for selection of pathogen-specific T cells out of heterogeneous hematological populations offered new possibilities leading to successful application of adoptive T cell transfer in HSCT patients with refractory virus (CMV, EBV, ADV) infections (152, 171, 186) (Tables 5–7). More recently adoptive cell transfer has been developed for other virus infections, like Varicella Zoster virus, BK virus, or human herpesvirus 6 (121, 187) as well as for invasive fungal infections with aspergillus or candida (135).

## Immunotherapy with Car Gene-Modified T Cells for Treatment of Leukemias

Despite the success of allogeneic HSCT in the quest for a cure of leukemic patients, the demand for alternative and new treatment options is high, as relapse and refractory leukemia remain a major challenge for patients having with very poor prognosis (188–190). How to improve the antitumor immunity, especially in patients who are not eligible for HSCT, need of a bridge therapy prior to transplant, or even after failure of HSCT. In the future, will there be a way even to replace SCT and thereby avoiding transplantation-associated complications?

Elimination of the malignant cells and sustained remissions can be achieved by induction of GvL effects after HSCT, which are based on a donor T cell-mediated immune response. Enhancement of the GvL effects is observed with DLI (191, 192). However, a treatment with the complete repertoire of allogeneic T cells is always accompanied by the substantial risk for the life-threatening GvHD. One way to increase anti-leukemic effects while avoiding GvHD in allogenic transplantation settings is the transfusion of *in vitro* selected T cells, specifically targeting tumor-associated antigens. But the majority of described tumor-associated antigens are not exclusively found in tumor cells, but represents self-antigens, either expressed in other adult healthy tissue or during embryonic development. In general, it is assumed the endogenous T cell repertoire against self-antigens show limited potency to eradicate tumor cells due to low affinity TCR. The most powerful T cells would target either neo-antigens derived from mutated genes within tumor cells or allogenic antigens like minor histocompatibility antigens with restricted expression in hematological cells, e.g., HA-1. These antigens are recognized as foreign proteins by the immune system (i.e., the T cell repertoire for these antigens is not shaped due to negative thymic selection of T cells expressing high-affinity TCRs). Another approach to break self-tolerance is the introduction of a new, high-affinity antigen specificity into the T cells, i.e., by genetic modification with an artificial TCR or with a CAR (12, 193).

### Clinical Outcome of CD19 CAR-Transduced T Cell Therapy

Recent success stories of therapy with CAR-modified T cells targeting CD19 in patients with high-risk B cell malignancies, such as chronic lymphocytic leukemia (CLL) or childhood acute lymphoblastic leukemia (ALL), have raised enormous scientific and public expectations. For example, in a clinical trial including 30 children and adults with relapsed or refractory ALL treated with CD19 CAR–transduced T cells 90% of the patients achieved complete remission (194). The development of CAR T cell therapy and a summary of clinical studies and data generated within the past years have been described in several reviews and therefore will not further discussed in this article (195, 196).

### Workflow of Adoptive Therapy with CAR-Engineered T Cells

**Figure 6:**
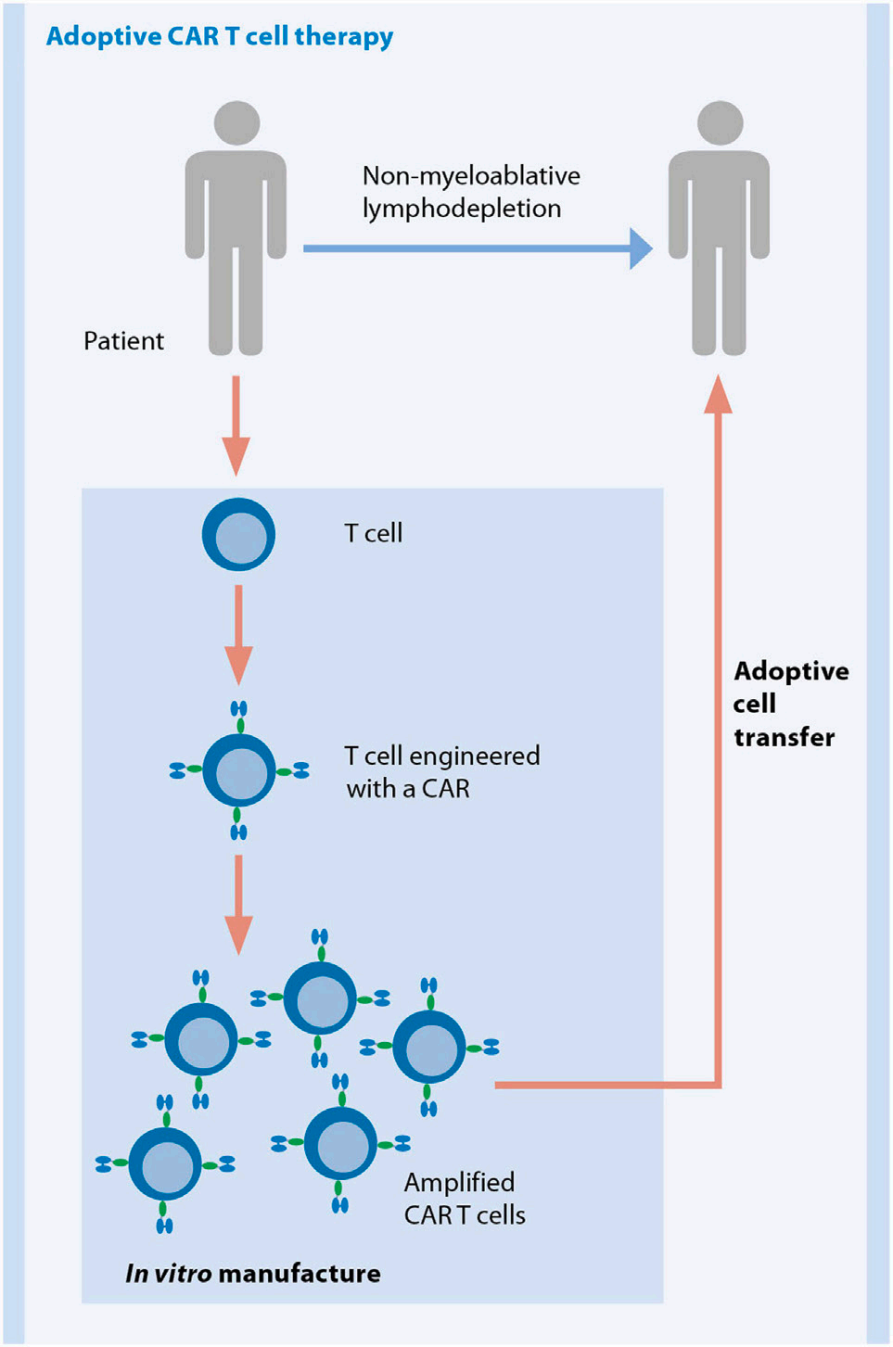
**General workflow for adoptive therapy with CAR-modified T cells**. Figure courtesy of Prof. Hinrich Abken.

To prepare CAR-modified T cells for the treatment of a leukemic patient, first peripheral blood is drawn from the patient. The T cells are then isolated from the blood and engineered *in vitro* with a CAR targeting a pre-defined antigen on tumor cells. Subsequently, the cells are amplified to obtain a sufficient number of CAR T cells for transfusion into the patient (Figure 6). Before administration of CAR T cells, the patient undergoes a non-myeloablative lymphodepletion, which supports the therapy, e.g., by promoting the *in vivo* proliferation and thus the persistence of CAR T cells.

### Engineering Potent and Safe CAR-Modified T Cells

**Figure 7:**
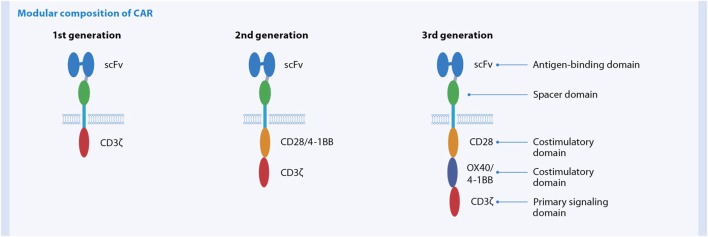
**Structure of different generations of CARs**. Figure courtesy of Prof. Hinrich Abken.

Chimeric antigen receptors are artificially constructed receptors introduced into somatic cells, mainly in T cells, by genetic engineering and redirect immune responses toward the tumor. A CAR consists of an extracellular antigen recognition motif, resembling a single-chain fragment of the variable region of an antibody (scFv), directed against a cell surface antigen expressed on a tumor cell (Figure 7). The scFv part is linked *via* a transmembrane domain to intracellular signaling structures derived from the TCR and costimulatory receptor(s). If CAR-engineered T cells encounter tumor-associated antigens, the intracellular signaling cascades of the TCR/costimulatory moieties are triggered. Ultimately, this activation results in T cell effector function, i.e., cell proliferation, cytokine secretion, and cytolytic activity (197). Over the last years, the functional properties of CARs have been improved. First-generation CARs lacked the intracellular signaling motifs for costimulation. Effective T cell activation requires at least two types of signals: (i) engagement of the TCR with antigen presented by MHC and (ii) engagement of costimulatory molecules, such as CD28, OX40, and 4-1BB. However, tumors often do not express appropriate ligands for costimulatory molecules. To overcome these restrictions second-generation CARs were developed incorporating the intracellular domains of one costimulatory receptor, either CD28 or 4-1BB. T cells expressing such CARs had a higher capacity to expand, mediate increased tumor killing, and persist *in vivo* for a longer period of time compared to first-generation CARs (198–201). With the aim to further improve the functionality of CAR-modified T cells, so-called “third-generation” CARs, which deliver more than one type of costimulatory signal, are now prepared for clinical trials.

Further efforts concentrate on strategies for design of T cells with the goal to overcome inhibitory T cell signaling, the suppression by the tumor microenvironment, or tumor antigen loss, which is now regularly detected in a subset of patients suffering from relapses after CD19 CAR T cell therapy (202–208).

Other strategies for CAR T cell design aim toward increasing the safety of CAR T cells. One major concern of the therapy is the attack of normal tissues (“on-target, off-tumor” toxicity), which could dependent on the chosen target antigen result in very severe and life-threating toxicity (208). However, the elimination of normal B cells with, e.g., CD19 CAR T cells and the resulting B cell aplasia is regarded as an expected and acceptable “on-target, off-tumor” effect, which is successfully treated with infusion of gamma immunoglobulins. Another toxicity first observed with CD19 CAR T cells is the cytokine release syndrome (202, 209, 210). It is a side-effect of the desired antitumor response induced by CAR T cells leading to mild, but in some cases to severe clinical syndromes, which requires intensive care and therapeutic management of the patients. Severe events are now effectively treated with blocking Anti-IL-6 receptor antibody without influencing the tumor rejection by the CAR T cells (211–213). Nevertheless, the need to prevent or substantially limit the toxicity of the therapy is high and potential solutions are under investigations (208, 214–216).

### Advantages of CAR-Engineered T Cells

Chimeric antigen receptors-modified T cells have some crucial advantages over natural T cells and in part also over TCR-engineered T cells, as they can function independently of MHC molecules. First, the affinity of an antibody–antigen binding is in general much higher compared to a TCR–peptide/MHC binding. This provides at least the option to target antigens that are usually not detected by T cells, e.g., carbohydrates and glycolipids, which are frequently altered in tumor cells (217–219). Second, loading of antigenic peptide onto MHC requires antigen processing and presentation, and both processes are targets of tumor escape mechanisms resulting in the loss of antigen/MHC expression on malignant cells. Third, a CAR recognizes its antigen independent of individual MHC allotypes, resulting in the universal application in all patients that express this antigen on the cell surface. In contrast a TCR is specific only for the combination of an antigenic peptide in the context with an MHC allele. Due to the MHC polymorphism in the human population, patient-specific or at least a panel of MHC allele/peptide-specific TCRs are needed to cover the human population comprehensively. Last, not only CD8^+^ T cells, but also CD4^+^ T cells can be engineered, which allows for T cell help independent of MHC class II expression. A clear disadvantage of CARs is that only cell surface antigens can be targeted, while intracellular tumor antigens remain invisible. However, the recognition of MHC/peptide complexes by CARs is not excluded (220), which might also facilitates access to intracellular tumor antigens.

As learned from the outcome of the clinical application of *ex vivo* expanded melanoma-infiltrating T cells over the last years, the key factors for a successful adoptive T cell therapy to target cancers are the selection of the best possible tumor antigen, the *in vivo* persistence of transferred T cells and their accessibility to the tumor. Beyond that, a reliable and reproducible manufacturing procedure leading to high-quality cellular products is a crucial element of the therapy (193). We will focus our discussion in the next sections on the demands and challenges connected to the manufacturing process and will disclose recent progress toward the implementation of therapy with CAR-engineered T cells into clinical practice.

### Complexity of the Cell Manufacturing Process

**Figure 8:**
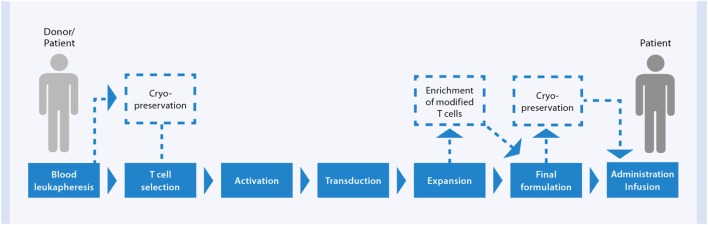
***In vitro* manufacture process of a CAR-engineered T cell product**.

Currently, therapies with CAR-modified T cells are mainly applied in the context of clinical trails by investigators, according to their own manufacturing process utilizing existing infrastructure with clean rooms, instruments etc. The *in vitro* preparation of CAR T cells is a quite complex process and lasts for several days to weeks. So far, most concepts for CAR T cell therapies are based on autologous cells, which means that each cellular product is manufactured in a single batch in small scale for a single patient. It starts with isolation of peripheral blood cells, e.g., by an initial leukapheresis step. Blood is drawn either from the patient directly (autologous therapy) or – in the case of a patient who received stem cell transplantation – from the stem cell donor (allogeneic therapy). Then T cells are enriched from the blood, activated, and subsequently gene-modified with viral or non-viral vectors encoding the CAR. The CAR-modified T cells are amplified to obtain larger numbers of cells and finally formulated and/or cryopreserved prior to infusion into the patient. Several in-process and quality control analyses of the cell product are required to guarantee the safety and quality of the final cellular end product (Figure 8). This multi-step workflow poses high demands on the infrastructure, is labor intensive, and requires various different techniques, devices, reagents, handling steps, and skilled and extensively trained operators. Within a small-scale clinical trial the entire process can be executed in a semi-automated manner with the use of several devices for single process steps according to GMP guidelines. To date only a restricted number of GMP facilities worldwide are able to carry out this manufacturing process. But in the light of the encouraging clinical outcomes, the need for a broadly applicable therapy is high. However, the transformation of such a manufacturing process into a routine and large-scale setting has some pitfalls. An optimization and an upscaling of the manufacturing process is one of the key factors for the dissemination of this therapy.

### Manufacture of High-Quality Cell Products Requires Robust and Reproducible Cell Processing

A favorable outcome of cell therapy depends on a robust and reproducible manufacturing processes resulting in safe and clinically effective cell products. Currently, many investigators and companies are developing solutions, including instruments, reagents, and consumables, for GMP-grade cell manufacture (221). Robustness of the cell manufacturing process, which will eliminate failure risks and allow standardization, can be improved by several means.

(1) Operation in a “closed” system in contrast to “open” processing minimizes the risk of contamination and therefore failure of production. Maintaining the sterility of the cell product is essential. All interventions during cell processing, like addition or exchange of reagents and buffer/media during washing, feeding, activation, transduction, and sampling steps bear the risk for product contamination. Closed systems are set-up with equipment that allows processing of cell without its exposure to the room environment, but keeping sterile conditions. Suitable closed systems are, e.g., bags with closed tubing pathways and connections. Introduction of material into closed systems is possible, e.g., *via* sterile filters. A suitable simplified and semi-closed cell culture system for CD19 CAR T cell production has been described (222). Closed systems might enable operators to work under less advanced GMP clean room conditions, which is more cost effective and easier to establish.
(2) The use of enriched T cells as starting material for the activation process helps to achieve higher reproducibility in the manufacturing process. Patient’s blood samples are highly variable in their cellular composition and one of the most critical parameter for reproducible cell processing. Instead of using the entire blood cell fraction for gene modification, isolation of T cells or even T cell subsets prior to modification is favorable for various reasons. Most patients are heavily pre-treated, which often give rise to abnormal or immunosuppressive blood cell populations or even low-responding T cells (223–225). Moreover, it has been shown that activation and expansion of the T cells is substantially enhanced when T cells were isolated from the blood product to eliminate suppressive influences (222, 226, 227). Currently, particular T cell subpopulations are under investigation with the aim to improve *in vivo* persistence and effector function of adoptively transferred CAR-modified T cells. One strategy is based on the modification of patient’s endogenous CMV- or EBV-specific T cell pools, which contain long-living memory cells (228). In addition, persistence of these CAR-modified T cells might be promoted by triggering the natural TCR *in vivo* upon reactivation of those latent viruses. A further advantage of CAR-modified virus-specific T cells is that they provide protection in the case of viral reactivation after lymphodepletion (228–230). A disadvantage of this concept is the need to implement the generation of virus-specific T cells into the manufacturing process, which adds more complexity to the whole process, is time consuming, and might affect functionality of the cells, especially if long-term culturing is required to obtain virus-specific T cell populations. Alternatively, the naive, central memory, or stem memory T cell subset, which have been described to have essential functional advantages, are regarded as an appropriate starting population (231–235).
  A straightforward and closed system for GMP-grade and large-scale T cell processing is the combination of Dynabeads^®^ CD3/CD28 CTS™, a large magnet (both offered by Thermo Fisher Scientific), and bags to enrich and concomitantly activate T cells from whole blood products (236). A versatile, reliable platform for closed, clinical-scale magnetic enrichment of either all T cell types or naive and central memory T cell subsets is the CliniMACS^®^ System, encompassing separation reagents and the CliniMACS Plus Instrument developed by Miltenyi Biotec, Bergisch Gladbach, Germany (237, 238). For T cell activation a reagent (MACS^®^ GMP TransAct™ CD3/CD28 Reagent) consisting of a biodegradable polymeric nanomatrix coated with agonists for CD3 and CD28 is available, which allows for efficient viral transduction (237). This reagent is in compliance with relevant GMP guidelines. It can be sterile filtered, which makes it a highly valuable tool for aseptic cell manufacturing.
(3) Simplification of the cell processing by automation improves reproducibility and reduces resources for operators and thus increases productivity. The CAR T cell manufacturing involves various process steps, like cell enrichment, cell culturing, final formulation of the product and in between cell washing, concentration, feeding, and rebuffering. In addition, in-process and quality controls samplings are performed. Several commercially available devices allow the run of single or few steps of the process (221). Nevertheless, multiple instruments and systems need to be implemented for execution of the whole process, which challenge the manufacturer in many ways. The different devices and the procedures need to be adjusted to each other to achieve a feasible and safe process. Substantial manual handling steps and user interactions are required. Additionally, each device demands installations, services, qualifications, and training of operators. A new device, the CliniMACS Prodigy^®^ instrument (Miltenyi Biotec), is designed as an all-in-one solution for automated cell processing in a closed GMP-compliant system (60). A process specifically developed and optimized for the manufacture of CAR T cells on this instrument is now available. With this process, the entire workflow for the manufacture of CAR T cells, starting with T cell enrichment through to final formulation, can be performed in a single-use tubing set with minimal operator interaction. The complex CAR T cell production process includes many different reagents, i.e., T cell separation reagents, activation and expansion reagents, viral vectors, cell culture media, cytokines, and buffers. Importantly, for the use of the CliniMACS Prodigy^®^ all these reagents are developed to efficiently and stably work together as an integrated reagent system. This CliniMACS Prodigy^®^ approach significantly simplifies the manufacturing process. In addition, due to the integrated solution it allows easy implementation in GMP facilities and can boost CAR T cell therapy to a standard-of-care.

### Future Perspectives: Commercialized Manufacture of Personalized Engineered Cellular Products

Today, most CAR T cell products are manufactured for phase I/II trials in a limited number either within clinical centers or facilities of commercial providers. At least with entering into phase II/III clinical trials new considerations have to be taken into account as the number of patients to be treated increases to hundreds or thousands per year. Production, infrastructure, and logistics for shipment of cellular materials have to be set up to guarantee the manufacturing of these high quantities in a high-quality and cost-effective manner and with compliance of all the regulatory requirements. To achieve these goals the process needs standardization and scale-up. In the end, a therapy must fulfill economical requirements to be available as a standard-of-care for patients.

Chimeric antigen receptors T cell therapy applied in the moment is cost-intensive as individualized products have to be generated starting with patient-derived cells. Several investigators are currently evaluating options to reduce the costs of cell production by depersonalizing T cell therapy, e.g., using off-the-shelf third-party T cells modified for knock-out of the endogenous HLA class I, TCR and/or CD52 expression for subsequent gene engineering with artificial antigen receptors (239, 240).

In principal, two different models for clinical cell manufacturing are discussed (221). A production line, as established for automated industries, where the manufacturing process for one patient product is structured in sequential operations, which are performed with specialized and dedicated personnel in physically separated spaces of the facility. In line with this concept is, e.g., the Xvivo modular laminar flow system from BioSpherix (Lacona, NY, USA), which enables the transport of cells through a whole series of areas. Due to the high investment for establishing a production line including the efforts required for organization of the infrastructure for cell shipments, a centralized manufacturing in highly specialized large facilities rather than a decentralized, local production at patients’ point-of-care is of favorite. The second model relies on devices such as the CliniMACS Prodigy^®^, to handle one cell sample in one instrument at a time in an automated way and with only a minimum of user interactions. Within one facility numerous devices can be run in parallel and completely independently from each other. The device-based system is in accordance with a centralized as well as a decentralized organized cell manufacturing and therefore an attractive solution for commercial providers having large or smaller facilities, including hospital located sites.

## Conclusion

Within the last years, the cellular immunotherapy field, especially in the context of hematological malignancies, gained tremendous attention by scientific researchers, clinicians, as well as commercial entities, thanks to the substantial progress made in multiple. The better scientific understanding of immunological mechanism and the novel advanced ideas and technologies for cell engineering and manufacturing have enabled the design of improved clinical approaches, which are currently being evaluated within clinical trials. The next step has to be the translation and broad implementation of these treatments into clinical routine. This requires on the one hand the selection of the best therapeutic options with maximal clinical benefit for the patients and on the other hand that the economical needs are met for all: the pharmaceutical companies and clinical entities involved in bringing the therapy to the patient, and the payers, who reimburse the therapy, i.e., health insurances.

## Ethics Statement

This study was carried out in accordance with the recommendations of “The Newcastle Hospitals NHS Foundation Trust Heath Research Authority-NRES Committee North East – Newcastle & North Tyneside 2” with written informed consent from all subjects. All subjects gave written informed consent in accordance with the Declaration of Helsinki.

## Author Contributions

All authors listed have made substantial, direct, and intellectual contribution to the work and approved it for publication.

## Conflict of Interest Statement

MQ was employed by Alcyomics, Ltd. at the time of writing. AD is Director of Alcyomics, Ltd. AR and LP are employees of Miltenyi Biotec GmbH. The other authors declare that the research was conducted in the absence of any commercial or financial relationships that could be construed as a potential conflict of interest.
